# miRNA regulated pathways in late stage murine lung development

**DOI:** 10.1186/1471-213X-13-13

**Published:** 2013-04-24

**Authors:** Sana Mujahid, Tanya Logvinenko, MaryAnn V Volpe, Heber C Nielsen

**Affiliations:** 1 Program in Cell, Molecular and Developmental Biology, Sackler School of Graduate Biomedical Sciences, Tufts University School of Medicine, Boston, MA, USA; 2 Institute for Clinical Research and Health Policy Studies, Tufts Medical Center, Boston, MA, USA; 3 Division of Newborn Medicine, Department of Pediatrics, Floating Hospital for Children at Tufts Medical Center, 800 Washington Street, Boston, MA, 02111, USA; 4 Department of Anatomy and Cell Biology Tufts University School of Medicine, Boston, MA, USA

**Keywords:** microRNA, Gestation, Sex, Profiling

## Abstract

**Background:**

MicroRNAs play important roles in regulating biological processes, including organ morphogenesis and maturation. However, little is known about specific pathways regulated by miRNA during lung development. Between the canalicular and saccular stages of the developing lung several important cellular events occur, including the onset of surfactant synthesis, microvascular remodeling and structural preparation for subsequent alveolarization. The miRNAs that are actively regulated, and the identity of their targets during this important developmental interval in the lung remain elusive.

**Results:**

Using TLDA low density real-time PCR arrays, the expression of 376 miRNAs in male and female fetal mouse lungs of gestational days E15 – E18 were profiled. Statistical analyses identified 25 and 37 miRNAs that changed significantly between sexes and with gestation, respectively. *In silico* analysis using Ingenuity Pathway Analysis (IPA) identified specific pathways and networks known to be targets of these miRNAs which are important to lung development. Pathways that are targeted by sex regulated miRNAs include retinoin, IGFR1, Tp53 and Akt. Pathways targeted by gestation-regulated miRNAs include VEGFA and mediators of glucose metabolism.

**Conclusion:**

MiRNAs are differentially regulated across time and between sexes during the canalicular and saccular stages of lung development. Sex-associated differential miRNA expression may regulate the differences in structural and functional male and female lung development, as shown by networks generated using *in silico* analysis. These data provide a valuable resource to further enhance the understanding of miRNA control of lung development and maturation.

## Background

It is clear that miRNAs regulate numerous important developmental processes in various organs. miRNAs are a class of small RNAs that post-transcriptionally regulate gene expression. The primary RNA (pri-miRNA) is transcribed from the genome and is processed by Drosha, an RNase III enzyme, into hairpin structured premature miRNAs (pre-miRNAs). These pre-miRNAs are exported out of the nucleus with the help of Exportin 5, and cleaved into double stranded duplexes by Dicer, another RNase III enzyme. The mature miRNAs are loaded onto nucleoprotein complexes called the RNA-induced silencing complex (RISC). The mature miRNAs in these complexes inhibit translation of target mRNAs by blocking translation or aiding in degradation of their targets [1].

An important role of miRNAs during lung morphogenesis has been recently established. Conditional deletion of Dicer from the lung epithelium led to arrested airway branching, dramatically altering lung organogenesis [2]. Subsequent studies of miRNA profile expression in the lung demonstrated that miRNAs are temporally expressed between the embryonic and adult lung development [3-7].

Mouse lung development is divided into five stages: embryonic (E9.5-E11.5), pseudoglandular (E11.5-16.5), canalicular (E16.5-E17.5), saccular (E17.5-P5) and alveolar (post-natal (P)5-P30). The coordination of multiple pathways across various cellular compartments is required to give rise to a fully developed lung. The transition from the late canalicular to early saccular stage is a crucial period in preparation for survival after birth. During the canalicular stage establishment of the air-blood barrier begins and epithelial differentiation of alveolar Type I and Type II cells commences. The onset of surfactant synthesis plus microvascular development in the early saccular stage are necessary for proper lung function at birth. The timing and coordination of genetic- and sex - specific programming that occurs during these lung developmental stages are crucial regulatory elements for lung maturation [8]. Very little is known about how miRNA expression and genetic control contributes to these critical events. The recent evidence showing the importance of miRNAs in development and disease of other organs indicates the need for more detailed studies of how miRNAs regulate pathways important in the developing lung [9].

The aim of this study was to analyze the dynamic regulation of miRNAs during the canalicular to early saccular stage of mouse lung development. We profiled miRNAs of lungs isolated from male and female E15-E18 fetal mouse lungs. It is well established that male infants have increased perinatal respiratory morbidity and mortality and that androgens inhibit lung maturation in males [10,11]. In mouse lung development, the development of surfactant production during this canalicular to early saccular interval is delayed by one day in males compared to females [12]. Several important transcription factors and signaling pathways are differentially expressed and activated in males and females during this time, suggesting that it is very likely that miRNAs may also be regulating these sex differences as well [13,14].

In this study, we show that a group of miRNAs are differentially expressed across these gestational ages, and another group of miRNAs differentially expressed between males and females. These groups were further analyzed for pathway interactions using Ingenuity Pathway Analysis, which maps interactive pathway networks based on established miRNA targets previously reported in the literature. With the IPA analysis we are able to highlight pathways regulated by these developmentally regulated miRNA groups that are differentially expressed across gestation, and between males and females, during an important window in lung development.

## Methods

The animal study protocol was approved by the Tufts Medical Center Institutional Animal Care and Use Committee. Principals of laboratory animal care were followed according to the National Institute of Health Guidelines for Care and Use of Laboratory Animals. Timed pregnant Swiss Webster mice were obtained from Charles River Laboratories (Wilmington, MA, USA), with the morning of the vaginal plug defined as gestational day 0 (E0).

### Isolation of total RNA

Timed pregnant mice at each gestational age were sacrificed using CO_2_ inhalation according to the approved protocol and in line with current regulations. The uterus was removed under sterile conditions by laparotomy and placed on ice. Fetuses were maintained in DMEM on ice while fetal sex was identified [15]. The lungs were removed *en block* under a sterile laminar airflow hood. Lungs were frozen for RNA extraction. RNA was extracted using miRVana miRNA Isolation Kit (Ambion, Grand Island, NY) according to the manufacturer’s instructions.

### miRNA arrays

A Taqman Array Rodent MicroRNA A card v2.0 (Applied Biosystems, Carlsbad, CA) was used to profile 376 mature miRNAs. cDNA was transcribed from total RNA using the Megaplex TM RT Rodent Primers Pool and the TaqMan MicroRNA Reverse Transcription Kit. The TaqMan miRNA Arrays were run on the ABI PRISM 7900 System using the TaqMan Universal PCR Master Mix according to the manufacturer’s instructions. Two array runs using separate lungs with two technical replicates per lung were performed. The data from these runs were used in the data analysis model. The delta CT was calculated by subtracting the miRNA Ct value from the Ct value of small nuclear U6 RNA, which served as a control. The data were normalized to E15 for data analysis. The data discussed in this publication have been deposited in NCBI’s Gene Expression Omnibus and are accessible through GEO Series accession number GSE46037 ( http://www.ncbi.nlm.nih.gov/geo/query/acc.cgi?acc=GSE46037).

### Data analysis

For each miRNA, we separately evaluated the effect of gestation (time) and gender (sex), and then tested if the temporal patterns differed between males and females. We used mixed linear models with fixed main time and sex effects, their interaction effect, and random sample effect to account for any correlated nature of the data. The analyses were performed using base, nlme, and qvalue packages of statistical software R [16-18].

Cluster analysis for the miRNAs that changed significantly across gestation and with fetal sex was done using gplots package of statistical R software [19]. Ingenuity Pathway Analysis (IPA) was used to generate pathways regulated by these miRNAs based on validated miRNA targets (Ingenuity Systems, http://www.ingenuity.com, August 2011 version).

## Results

### Specific miRNAs are differentially expressed between sexes and with advancing gestation

MiRNA profiles from E15-E18 male and female mouse lungs were generated using a miRNA qPCR array (Additional file 1). Using linear mixed models we identified those miRNAs that changed significantly with gestation or with sex (p<0.05, FDR<33%). This analysis identified 37 miRNAs that significantly changed with advancing gestation and 25 miRNAs that were significantly different between males and female fetuses. A group of 13 miRNAs changed significantly with sex and gestation (interactive effect, Additional file 2). The majority of the 13 miRNAs with an interactive effect were also significant by either gestation or sex. The miRNAs that changed significantly with gestation and sex are listed in Table 1, along with their p-values, chromosomal locations, the miRNA family, and miRNA cluster that shows the number of other miRNAs located within the neighboring 10 kb chromosomal region. Fold change values are listed in Additional file 3.

**Table 1:** miRNAs that changed significantly with sex and gestation

	**miRNA gene**	**P-value**	**Chromosome**	**Chromosomal location**	**miRNA family**	**miRNA cluster**
*Sex*						
	mmu-miR-802	0.025	16	intergenic	mir-802	
	mmu-miR-138	0.047	9,8	intergenic	mir-138	
	mmu-miR-182	0.026	6	intergenic	mir-182	
	mmu-miR-296-3p	0.034	2	intergenic	mir-296	1
	mmu-miR-125a-5p	0.037	17	exon	mir-125	2
	mmu-miR-532-3p	0.002	X	intron	mir-188	3
	mmu-miR-652	0.04	X	intron	mir-652	
	mmu-miR-455	0.01	4	intron	mir-455	
	Y1	0.002	*	*	*	
	mmu-miR-670	0.016	2	intron, exon	mir-670	
	mmu-miR-367	0.008	3	intron	mir-367	4
	rno-miR-207	0.013	5	intron	mir-207	
	mmu-miR-31	0.001	4	intergenic	mir-31	
	rno-miR-351	0.018	X	intergenic	mir-351	
	mmu-miR-351	0.009	X	intergenic	mir-351	
	mmu-miR-220	0.013	*	*	*	
	mmu-miR-219	0.015	17,2	exon, intergenic	mir-219	
	mmu-miR-24	0.013	13,8	intron, exon	mir-24	
	mmu-miR-141	0.04	6	intron	mir-8	
	rno-miR-743b	0.006	X	intergenic	mir-743	3
	mmu-miR-470	0.005	X	intergenic	mir-743	1
	mmu-miR-615-3p	0.001	15	intron	mir-615	
	rno-miR-327	0.002	*	*	mir-327	
	mmu-miR-742	0.002	X	intergenic	mir-742	4
	mmu-miR-486	0.035	8	intron	mir-486	1
*Gestation*						
	mmu-miR-452	0.011	X	intron	mir-452	1
	mmu-miR-147	0.031	2	exon	mir-147	
	mmu-miR-504	0.018	X	intron	mir-504	
	rno-miR-743b	0.005	X	intergenic	mir-743	3
	mmu-miR-470	0.005	X	intergenic	mir-743	1
	mmu-miR-409-3p	0.011	12	exon	mir-154	13
	mmu-miR-92a	0.027	14,X	exon, intergenic	mir-25	5
	mmu-miR-17	0.039	14	exon	mir-17	5
	mmu-miR-18a	0.008	14	exon	mir-17	5
	mmu-miR-670	0.039	2	intron, exon	mir-670	
	mmu-miR-367	0.011	3	intron	mir-367	4
	rno-miR-760-5p	0.023	2	intergenic	mir-760	
	mmu-miR-325	0.025	X	intron	mir-325	
	mmu-miR-220	0.006	*	*	*	
	mmu-miR-351	0.039	X	intergenic	mir-351	6
	mmu-miR-200c	0.003	6	intron	mir-8	1
	mmu-miR-484	0.019	16	Intergenic	mir-484	
	mmu-miR-24	0.007	13,8	intron, exon	mir-24	3
	mmu-miR-126-3p	0.04	2	intron, exon	mir-126	
	mmu-miR-26a	0.008	9, 10	intron	mir-26	1
	mmu-miR-30e	0.015	4	intron	mir-30	2
	mmu-miR-322	0.002	X	intergenic	mir-322	6
	mmu-miR-146a	0.001	11	intergenic	mir-146	
	mmu-miR-150	1x10^-3^	7	intergenic	mir-150	1
	mmu-miR-34b-3p	1x10^-3^	9	intergenic	mir-34	1
	mmu-miR-29a	0.027	6	intergenic	mir-29	1
	mmu-miR-30d	1x10^-3^	15	exon	mir-30	1
	mmu-miR-146b	0.017	19	intron	mir-146	
	mmu-miR-27a	0.017	8	intergenic	mir-27	3
	mmu-miR-30a	1x10^-3^	1	intergenic	mir-30	
	mmu-miR-139-3p	0.005	7	intron	mir-139	
	mmu-miR-615-3p	0.002	15	intron	mir-615	
	rno-miR-327	0.004	*	*	mir-327	
	mmu-miR-742	0.003	X	intergenic	mir-742	4
	mmu-miR-486	0.007	8	intron	mir-486	1
	mmu-miR-340-5p	0.049	11	intron	mir-340	
	mmu-miR-449a	0.025	13	intron	mir-449	2

miRNAs that changed significantly with sex and gestation are listed, along with their p-value, chromosome number, the region in which they are located, the miRNA family they belong to, and the number of miRNAs located within the cluster.

*Information not available.

### Networks for miRNAs changing significantly with sex

**Figure 1:**
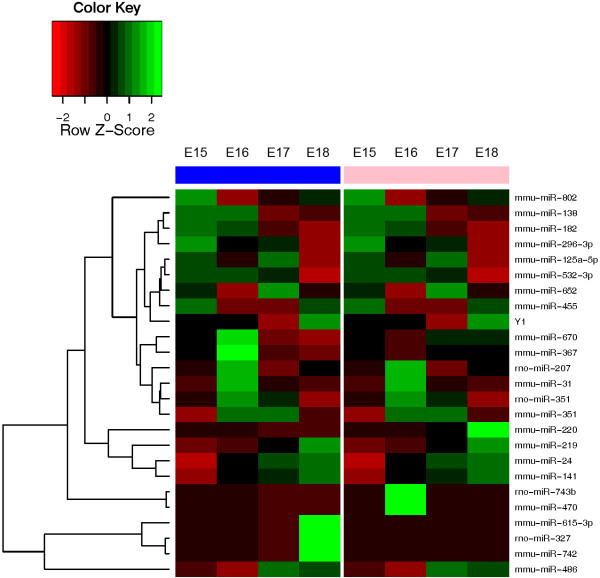
**Expression patterns of miRNAs that changed significantly with sex between E15 – E18 male and female lungs.** Total RNA was isolated from male and female E15 – E18 whole lungs and miRNA expression profiling was done using Taqman Rodent miRNA real-time PCR array. Columns with blue bars indicated male lungs, and columns with pink bars indicate female lungs. Each gestational day is normalized to male E15 time point. Black (0) represents mean value for a gene, green indicates the expression above the mean, and red represents expression below the mean, as shown in the color key.

**Figure 2:**
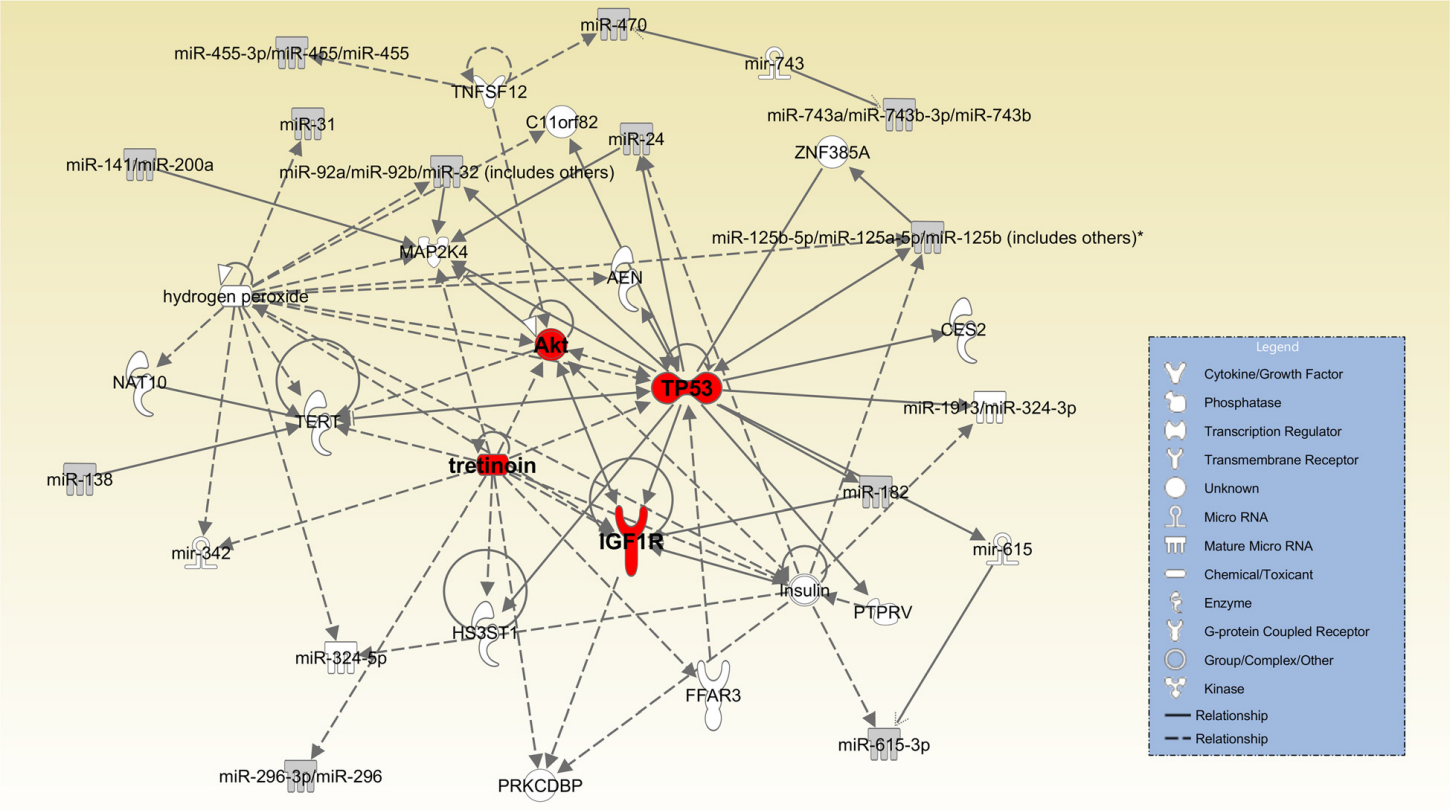
**Network associated with miRNAs that were significantly different between males and females*.*** IPA was used to generate a network of molecules that are validated targets of miRNAs shown in Figure 1. This network converges on retinoin, IGFR1, Akt and p53. Solid lines indicate known relationships. Dashed lines indicate proposed relationships.

The cluster dendrogram for miRNAs that changed significantly between the development of male and female lungs is shown in Figure 1. Sex hormones have been shown to regulate lung development. Androgens, in particular, have inhibitory effects on lung maturational events during the gestational interval studied [20-23]. Some of the identified miRNAs have similar gestational trends between males and females, but the absolute levels of miRNAs differed greatly between the sexes (Additional file 1, Additional file 3). In order to decifer the known functions of the sex-regulated miRNAs, Ingenuity Pathway Analysis software was used to generate molecular pathways based on known targets of the respective miRNAs. IPA identified two different yet related networks for those miRNAs significantly different in developing lung by sex, based on previously validated targets in the literature (Figure 2). The first network converged upon several central molecules, only some of which have been studied in the context of lung development. The central convergent molecules include retinoin, IGF1R, Akt and Tp53.

Retinoic acid, a metabolite of retinoin, promotes lung maturation and inhibits androgen receptor levels and function in several organs [24-27]. Further, testosterone has been shown to modulate retinoic acid activity in the lacrimal gland [28]. miR-296-3p, miR-125a-5p, miR-342 and miR-486 have all been shown to be regulated by retinoic acid (Figure 2; Additional file 4). IGF signaling has also been shown to positively influence lung maturation and vascularization, processes that are negatively regulated by androgens [29]. Futhermore, IGF1R may facitilate signaling of other growth factors known to affect fetal lung maturation in a sex-specific fashion, such as EGFR [12,30]. miR-182 can regulate IGF1R expression (Figure 2; Additional file 4). IGFs have the ablity to regulate proliferation and differentiation, processes that play an important role in late lung development and account for sex differences. IGF1R total protein levels increase dramatically between gestational day 16.5 and 17.5, right at the onset of surfactant synthesis [31,32]. Given our miRNA data, specific miRNAs can be linked with sex-specific changes in retinoic acid, androgen control and IGF signaling over an interval in lung development in which male infants are known to have a clinical disadvantage in disease related to delayed lung maturation.

Akt plays several important roles in development through control of cell proliferation, migration, apoptosis and transcription. Many of the differentially expressed miRNAs are involved in cellular responses to amino acid, growth factor, and hormonal stimuli based on GO Annotations of these miRNAs (Additional file 4). Several signaling molecules and receptors that are important in lung maturation, including EGFR, ErbB4, PDGF and insulin, activate Akt signaling. Of these, only EGFR is known to have a sex-specific development of expression and function in fetal lungs during this window of development [33,34]. Pharmacological manipulations of Akt have also been shown to affect surfactant synthesis synthesis [35].

Other focal centers in the network include Tp53 and MAP2K4, both of which are tumor suppressor genes that are deregulated in lung cancer [36,37]. In particular, miR-486, miR-24, miR-182, miR-615-3p and miR-125a-5p are regulated by Tp53 (Figure 2; Additional file 4). Little is known about the functions of Tp53 and MAP2K4 during lung development, though it is thought that p53 may be involved in alveolar epithelial cell differentiation [38].

**Figure 3:**
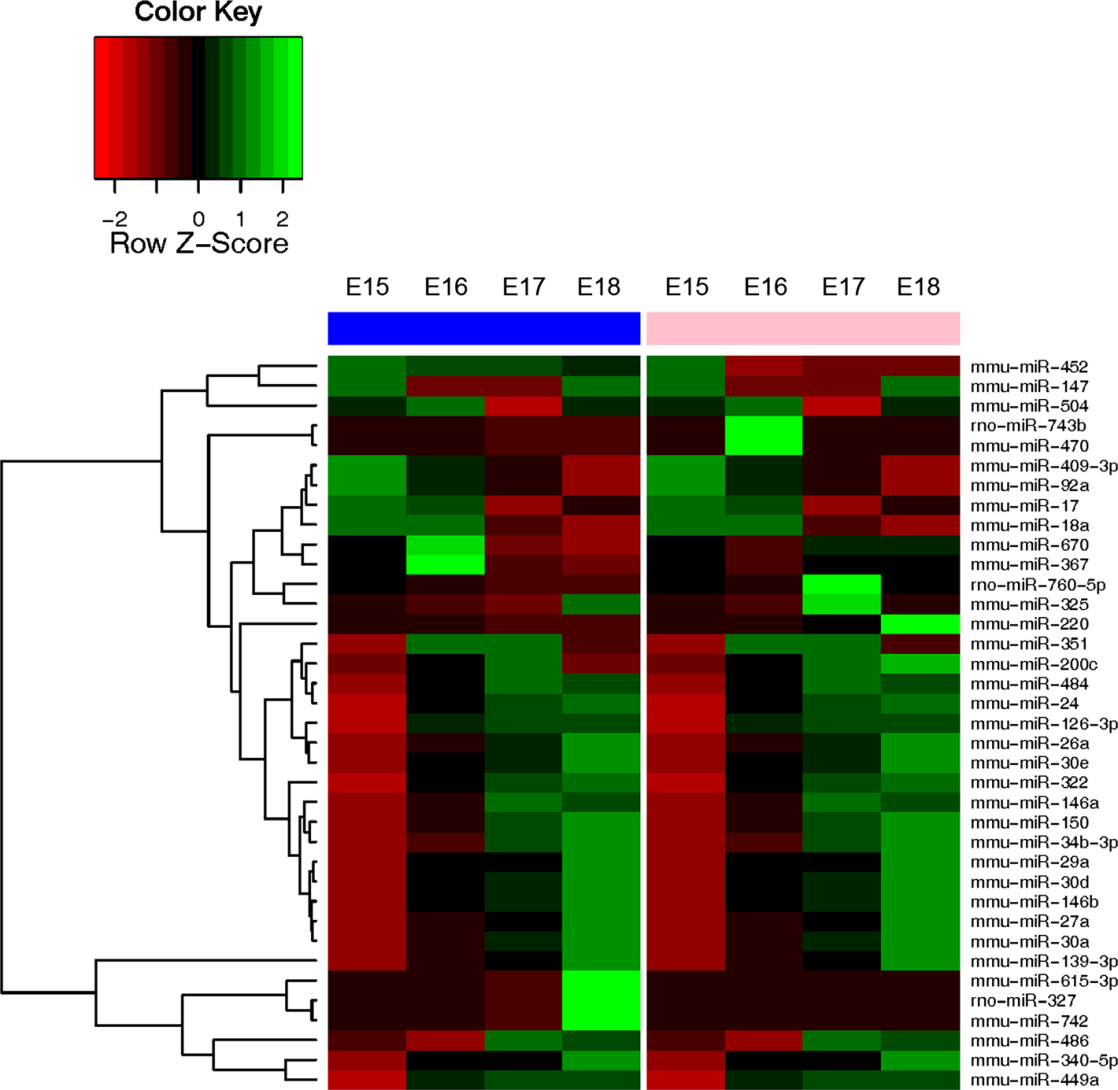
**Expression patterns of miRNAs that changed significantly with gestational E15 – E18 lungs.** Total RNA was isolated from male and female E15 – E18 whole lungs and miRNA expression profiling was done using Taqman Rodent miRNA real-time PCR array. Columns with blue bars indicated male lungs, and columns with pink bars indicate female lungs. Each gestational day is normalized to male E15 time point. Black (0) represents mean value for a gene, green indicates the expression above the mean, and red represents expression below the mean as shown in the color key.

### Networks for miRNAs changing significantly with gestation

**Figure 4:**
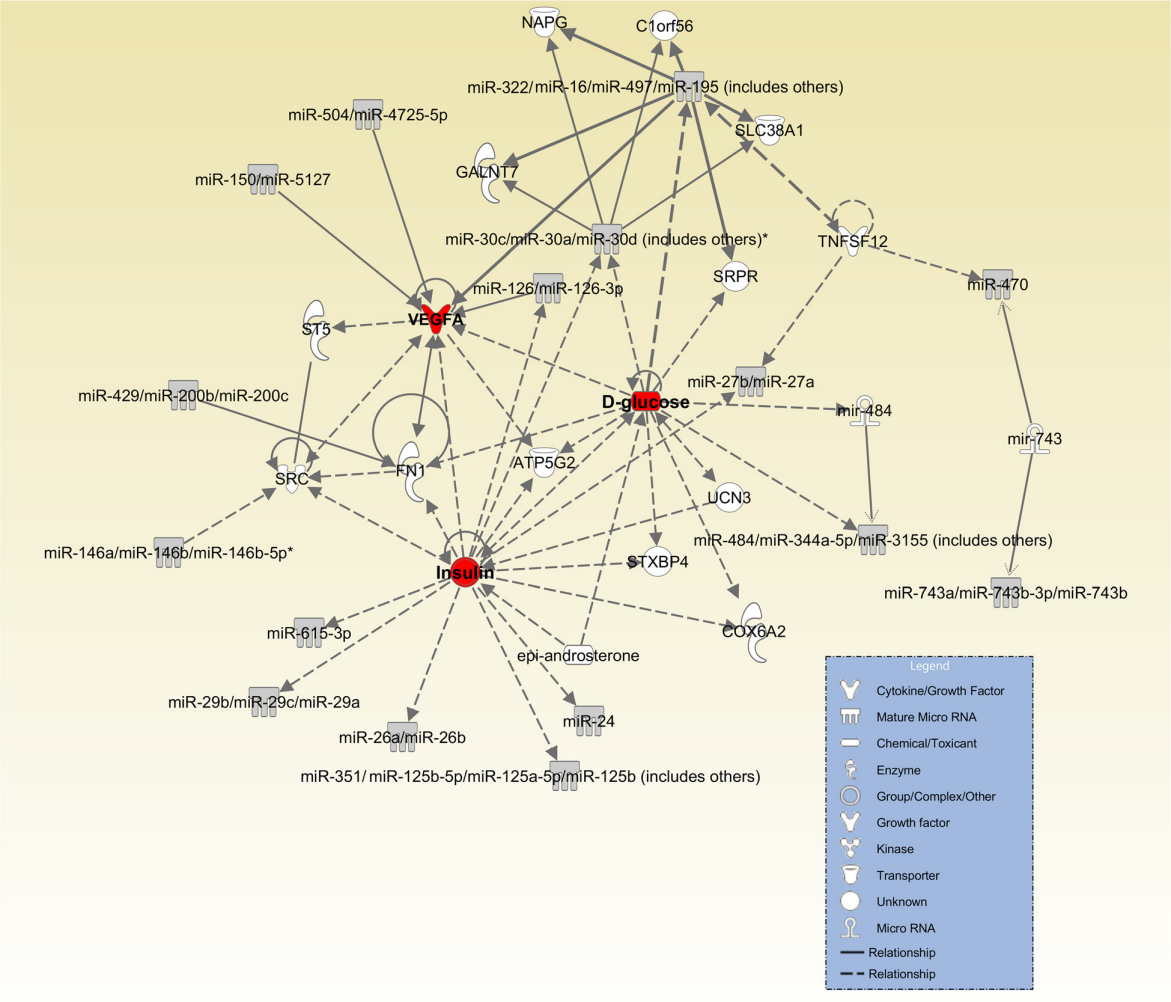
**Network associated with miRNAs that were significantly different across gestation.** IPA was used to generate a network of molecules that are validated targets of miRNAs shown in Figure 4. This network converges on IGF, d-glucose and VEGFA. Solid lines indicate known relationships. Dashed lines indicate proposed relationships.

The cluster dendrogram for miRNAs that changed significanty with gestation is shown in Figure 3. The two central components of this network, based on previously established targets as identified by IPA analysis, are mediators of glucose metabolism (regulation of d-glucose production and of insulin production) and vascular endothelial growth factor A (VegfA) (Figure 4). Insulin binding to the insulin receptor (IR) regulates glucose uptake and metabolism, which is required to produce substrate for surfactant phospholipid synthesis [39]. The IR, which is mainly expressed in the alveolar Type II cells, is developmentally regulated in its expression [39,40]. Several of these differentially expressed miRNAs are associated with the insulin pathway. These include miR-24, miR-30e, miR-351, miR-26a and miR-27a (Figure 4; Additional file 4).

VEGFA helps coordinate lung epithelial and endothelial maturation. It is produced and secreted in late gestion lung by the alveolar epithelium and acts by binding to receptors on the vascular endothelium in the adjacent mesenchyme [41]. As the lung progresses from the canalicular to saccular stage of development, VEGF levels increase as the alveolar sacs form and an air-blood interface becomes established. Further, several studies have shown that VEGFA mRNA expression increases towards the end of gestation [3,42]. As shown by transgenic and knockout models, VEGFA levels are tightly regulated during lung development. Both increased and decreased expression of this factor is severely detrimental to vascular development, including lung vascular development, where it impacts saccular, alveolar and microvasculature development within the alveolar unit [43-45]. miR-126, in particular, enhances the proangiogenic effects of VEGF and FGF, by targeting SPRED1 [46]. Several other miRNAs, such as miR-150, miR-504 and miR-322 have all been shown to regulate VEGFA expression (Figure 4).

Several other molecules with potential significance for lung development were also identified by the IPA analysis. These include fibronectin 1, Src, regulators of neurotransmitter function (NAPG, SLC38A1), regulators of protein synthesis and processing (GLANT7, SPRP), and those that control mitochondrial function (COX6Af2, ATPSG2). Of these, fibronectin has been most studied in lung development. Its expression is temporally regulated and helps to stabilize newly formed airways [47,48].

## Discussion

The importance of miRNA control of gene expression through regulation of mRNA translation during fetal organ development is becoming increasingly recognized. MiRNAs regulate specific elements of cardiac morphogenesis, brain development, and hematologic development [49-51].

Previous profiles of miRNA expression in lungs have been published [3-7]. Overall, such studies have analyzed single time points across a wide span of the different stages of development and maturation. Our study adds to that information by providing a focused analysis of a specific window of development, specifically the progression from late pseudoglandular through the canalicular and further into the early saccular development stages. This is a crucial developmental period during which the lung becomes capable of the normal cellular and physiologic functions necessary for gas exchange to support life after birth. Dominant features of this crucial developmental interval are the development of Type II epithelial cell maturation for surfactant synthesis, the establishment of the microvascular network responsible for efficient gas exchange, and the thinning of the interstitial mesenchyme to promote an increase in epithelial surface area for efficient gas exchange. Using data from this array, we have gone on to study the role of specific miRNAs in lung airway and vascular development [52].

A major challenge for miRNA studies, whether profiling the microRNAome or studying an individual miRNA, is the identification of specific target proteins whose expression is mediated by specific miRNAs. Several valuable algorithms have been developed which predict miRNA targets based on the physical chemistry and codon sequences of mRNA molecules [53]. Such algorithms commonly predict from tens to literally hundreds of potential targets for a given miRNA. Not surprisingly, the overwhelming majority of such predicted target molecules remain unvalidated. An additional difficulty is that such algorithms predict different targets, based on the parameters used to develop the algorithm. A pathway analysis tool such as IPA is a powerful tool for analyzing interactive networks involving multiple miRNA molecules based on previously established targets. By focusing the pathway analysis on those miRNAs which changed significantly during the interval studied, important clues can be obtained to direct subsequent focused studies of particular developmental events in order to identify specific targets.

Our analysis shows that of the large number of common miRNAs expressed in the mouse, only a relatively small number show significant developmental or sex-specific regulation during the interval studied. This suggests that miRNA targets are likely to be focused on processes central to the preparation for extrauterine life. Several of the core elements identified by the pathway analysis substantiate this conclusion. Molecules significant for vascular development, structural remodeling, cell metabolism, and cell proliferation and differentiation were prominent in the analytical trees. A particular value of the pathway analysis is that it facilitates identification of specific miRNAs that have been shown to be involved in these processes, making them a good starting point to be studied in the context of lung development. By focusing on miRNAs known to participate in specific processes (e.g. miR-126 in vascular formation), one can begin to tease out their roles in the developing lung.

The idea that miRNAs have sex dependent expression patterns is not new. In fact, miRNAs show sex-specificity during Embryonic Stem (ES) cell differentiation [54]. Male and female differences in physiology and behavior are widely recognized as contributors to disparities in health outcomes. miRNAs are postulated to play an important role in the sexually dimorphic brain [55]. Sex-based miRNA differences can also contribute to cardiac pathologies [56,57]. Our data suggest that miRNAs may regulate sex differences in the lung. The role of androgens in regulating lung sex-specific differences during development has been well established [13]. In light of this information it is likely that many of these miRNAs are androgen regulated and further studies would enable identification of androgen regulated miRNA pathways in the lung. However, it is possible that not all of the sex-specific miRNAs are regulated by androgens. Other factors, such as estrogen and TGFβ, have also been shown to contribute to sex-specific differences in lung [20].

A few of the miRNAs that were identified as significant in our analysis have been studied by other groups. Transgenic overexpression of the miR17-92 cluster altered proliferation and differentiation of lung progenitor cells, and Rbl2 was identified as a target of miR17-5p [58]. A subsequent study showed that the miR-17 family can target Stat3 and Mapk14 to modulate FGF10-FGFR2b signaling, which is important in lung epithelial branching morphogenesis [59]. The miR-302/367 cluster has been shown to regulate the balance between lung progenitor cell proliferation and differentiation, as well as progenitor cell polarity [60].

The results from this study should not be interpreted to indicate that overall only a few miRNAs are active in the control of lung development. It is highly probable that many molecules are under miRNA regulation which does not change within the interval studied. This is evident when our results are analyzed within the context of other studies which evaluated lungs at time points outside of the focus of our study. Our work does illustrate the value of a focused analysis to identify function-oriented targets of miRNA regulation, based on what is known about these specific miRNAs.

## Conclusions

We have identified a set of miRNAs which exhibit either developmental or sex-specific expression differences during a crucial interval of lung development in preparation for birth. Pathway analysis identifies convergent interactive signaling networks that permit subsequent studies of focused identification and analysis of miRNA-target relationships, based on previously established targets. This has significant value in determining the role of miRNAs for development of the fetal lung.

## Abbreviations

miRNA: microRNA; RISC: RNA induced silencing complex; IGFR1: Insulin-like growth factor receptor 1; IGF: Insulin-like growth factor; Akt: Protein Kinase B; Tp53: Tumor protein 53; EGFR: Epidermal growth factor receptor; PDGF: Platelet-derived growth factor; VEGFA: Vascular endothelial growth factor A; IR: Insulin receptor; Src: Sarcoma

## Competing interests

The authors declare that they have no competing interests of a financial or non-financial nature.

## Authors’ contributions

SM participated in the design of the study, carried out the experiments, performed the IPA analysis and participated in writing the manuscript. TL performed the array analysis and developed the heat map figures. MVV participated in the design of the study and in writing the manuscript. HCN participated in the design of the study and in writing the manuscript. All authors read and approved the final manuscript.

## Authors’ information

MaryAnn V Volpe and Heber C Nielsen are co-senior authors.

## Supplementary Material

Additional file 1 **Figure:*miRNA profiling of male and female lungs.*** Total RNA was isolated from male and female E15 – E18 whole lungs and miRNA expression profiling was done using Taqman Rodent miRNA real-time PCR array. 375 miRNAs were profiled and Ct values were normalized to U6 snRNA house keeping gene. The technical and biological replicates were averaged. Click here for file

Additional file 2 **Figure:*Expression patterns of miRNAs that changed significantly with sex and gestation.*** Total RNA was isolated from male and female E15 – E18 whole lungs and miRNA expression profiling was done using Taqman Rodent miRNA real-time PCR array. Columns with blue bars indicated male lungs, and columns with pink bars indicate female lungs. Each gestational day is normalized to the male E15 time point. mmu-miR-363 and mmu-miR-325 are the only miRNAs that are exclusive to this interaction group. The rest of the miRNAs in this group overlap with those present in the sex and gestation groups. Click here for file

Additional file 3 **Table:*List of miRNA regulators and targets.*** Using the Ingenuity Pathway Analysis database, the GO Annotations, upstream regulators, and downstream regulators/targets of miRNAs that changed significantly between sexes and with gestation were identified. Click here for file

Additional file 4 ***Fold change values of miRNAs that changed significantly between sexes and gestation.*** Fold change values were calculated using deltaCT values in Additional file 1. Click here for file
