# Supplementary material for: miRNA regulated pathways in late stage murine lung development
Source: BMC Dev Biol. 2013 Apr 24;13:13. doi: 10.1186/1471-213X-13-13 (PMC3644234; doi:10.1186/1471-213X-13-13)
Supplement: Additional file 1 — Figure:miRNA profiling of male and female lungs. Total RNA was isolated from male and female E15 – E18 whole lungs and miRNA expression profiling was done using Taqman Rodent miRNA real-time PCR array. 375 miRNAs were profiled and Ct values were normalized to U6 snRNA house keeping gene. The technical and biological replicates were averaged. [file 1471-213X-13-13-S1.docx]

|  | M15 | M16 | M17 | M18 |  | F15 | F16 | F17 | F18 |
| --- | --- | --- | --- | --- | --- | --- | --- | --- | --- |
| miRNA | Delta Ct values | | | |  | Delta Ct values | | | |
| mmu-let-7a | 10.075 | 9.083 | 9.523 | 14.566 |  | 15.122 | 15.035 | 14.784 | 14.891 |
| mmu-let-7b | 8.740 | 8.529 | 9.078 | 7.932 |  | 8.591 | 8.570 | 8.422 | 7.949 |
| mmu-let-7c | 8.654 | 8.634 | 9.008 | 8.112 |  | 9.084 | 8.851 | 8.276 | 7.995 |
| mmu-let-7d | 9.184 | 8.474 | 8.887 | 7.692 |  | 8.775 | 8.810 | 8.073 | 7.973 |
| mmu-let-7e | 7.142 | 6.531 | 6.827 | 5.945 |  | 7.268 | 6.691 | 7.481 | 5.933 |
| mmu-let-7f | 10.779 | 10.112 | 10.581 | 15.237 |  | 15.641 | 15.880 | 15.406 | 15.649 |
| mmu-let-7g | 10.524 | 9.649 | 10.039 | 8.645 |  | 9.830 | 9.681 | 8.726 | 9.040 |
| mmu-let-7i | 9.833 | 9.104 | 9.302 | 7.937 |  | 9.025 | 8.834 | 8.621 | 7.885 |
| mmu-miR-100 | 10.637 | 10.617 | 11.314 | 11.037 |  | 10.268 | 10.524 | 11.251 | 10.437 |
| mmu-miR-101a | 12.895 | 12.415 | 12.850 | 12.548 |  | 11.721 | 12.303 | 15.759 | 11.904 |
| mmu-miR-103 | 14.140 | 18.305 | 11.668 | 12.793 |  | 19.438 | 12.176 | 23.816 | 11.531 |
| mmu-miR-105 | 24.049 | 20.890 | 28.478 | 19.885 |  | 22.990 | 11.775 | 18.295 | 20.121 |
| mmu-miR-106a | 5.662 | 5.702 | 6.385 | 6.000 |  | 5.248 | 5.580 | 6.764 | 6.270 |
| mmu-miR-106b | 10.506 | 9.973 | 10.543 | 9.740 |  | 9.335 | 9.581 | 10.525 | 9.759 |
| mmu-miR-107 | 15.533 | 15.020 | 15.340 | 16.256 |  | 15.384 | 17.612 | 15.045 | 14.422 |
| mmu-miR-10a | 10.722 | 11.201 | 11.232 | 10.896 |  | 10.562 | 11.127 | 11.619 | 10.998 |
| mmu-miR-10b | 19.853 | 14.528 | 16.750 | 17.099 |  | 17.804 | 18.090 | 17.676 | 17.442 |
| mmu-miR-122 | 14.085 | 14.710 | 13.795 | 15.691 |  | 17.001 | 18.643 | 20.848 | 12.582 |
| mmu-miR-124 | 25.304 | 21.286 | 26.114 | 20.047 |  | 21.941 | 22.483 | 20.972 | 24.147 |
| mmu-miR-125a-3p | 15.531 | 15.527 | 15.940 | 17.417 |  | 16.407 | 14.985 | 13.592 | 15.126 |
| mmu-miR-125a-5p | 7.282 | 7.567 | 7.092 | 8.667 |  | 8.611 | 8.880 | 13.193 | 8.215 |
| mmu-miR-125b-3p | 20.440 | 17.939 | 19.594 | 19.370 |  | 18.998 | 19.490 | 19.306 | 20.597 |
| mmu-miR-125b-5p | 9.096 | 9.530 | 9.774 | 10.261 |  | 9.358 | 9.805 | 8.540 | 9.247 |
| mmu-miR-126-3p | 5.034 | 3.709 | 3.674 | 4.292 |  | 5.286 | 4.556 | 5.031 | 3.454 |
| mmu-miR-126-5p | 9.675 | 8.523 | 8.520 | 8.569 |  | 9.545 | 9.106 | 8.418 | 7.917 |
| mmu-miR-127 | 8.842 | 8.887 | 8.827 | 8.369 |  | 8.313 | 8.535 | 9.985 | 7.682 |
| mmu-miR-128a | 15.715 | 15.081 | 16.076 | 17.502 |  | 14.978 | 15.254 | 18.354 | 17.050 |
| mmu-miR-129-3p | 17.150 | 16.662 | 17.251 | 19.700 |  | 18.469 | 17.357 | 21.300 | 19.069 |
| mmu-miR-129-5p | 27.569 | 23.806 | 23.513 | 18.620 |  | 21.676 | 21.858 | 18.935 | 19.461 |
| mmu-miR-130a | 9.885 | 9.429 | 9.603 | 9.313 |  | 9.332 | 8.862 | 9.165 | 8.892 |
| mmu-miR-130b | 10.739 | 11.014 | 11.776 | 11.566 |  | 10.283 | 10.326 | 10.520 | 11.069 |
| mmu-miR-132 | 11.107 | 10.928 | 11.310 | 16.311 |  | 15.570 | 15.679 | 14.633 | 15.905 |
| mmu-miR-133a | 7.045 | 4.870 | 8.441 | 6.131 |  | 6.360 | 7.301 | 8.887 | 6.380 |
| mmu-miR-133b | 11.160 | 9.661 | 13.083 | 10.829 |  | 10.299 | 11.737 | 11.414 | 10.326 |
| mmu-miR-134 | 8.300 | 8.675 | 8.791 | 9.553 |  | 8.165 | 8.286 | 10.047 | 9.017 |
| mmu-miR-135a | 16.688 | 16.156 | 16.257 | 16.815 |  | 16.103 | 17.534 | 14.917 | 17.457 |
| mmu-miR-135b | 11.345 | 10.518 | 10.925 | 11.334 |  | 11.003 | 10.599 | 10.303 | 10.323 |
| mmu-miR-136 | 12.273 | 10.949 | 11.323 | 10.359 |  | 10.740 | 10.543 | 11.501 | 9.591 |
| mmu-miR-137 | 16.614 | 16.506 | 17.576 | 16.161 |  | 16.100 | 16.329 | 17.437 | 15.927 |
| mmu-miR-138 | 12.330 | 12.004 | 13.336 | 12.996 |  | 11.068 | 11.426 | 12.223 | 12.088 |
| mmu-miR-139-3p | 24.063 | 19.689 | 15.641 | 15.496 |  | 23.940 | 19.958 | 16.063 | 16.202 |
| mmu-miR-139-5p | 10.562 | 9.699 | 9.870 | 10.416 |  | 10.896 | 10.143 | 10.020 | 10.050 |
| mmu-miR-140 | 9.937 | 8.919 | 9.597 | 9.205 |  | 9.346 | 8.727 | 9.208 | 8.216 |
| mmu-miR-141 | 13.916 | 12.386 | 12.668 | 11.688 |  | 12.389 | 11.591 | 10.732 | 10.731 |
| mmu-miR-142-3p | 11.647 | 10.491 | 11.026 | 10.748 |  | 10.359 | 10.312 | 11.527 | 9.564 |
| mmu-miR-142-5p | 16.806 | 15.953 | 15.997 | 15.880 |  | 16.196 | 16.936 | 13.364 | 14.985 |
| mmu-miR-143 | 10.141 | 9.506 | 9.787 | 8.302 |  | 9.534 | 9.451 | 10.620 | 8.524 |
| mmu-miR-1 | 12.355 | 10.214 | 14.679 | 12.101 |  | 11.977 | 12.883 | 11.921 | 12.549 |
| mmu-miR-145 | 6.210 | 6.137 | 6.063 | 6.558 |  | 6.482 | 6.309 | 6.417 | 5.525 |
| mmu-miR-146a | 9.695 | 8.024 | 7.139 | 7.933 |  | 9.391 | 8.854 | 8.163 | 6.987 |
| mmu-miR-146b | 12.084 | 10.875 | 10.404 | 10.261 |  | 11.928 | 11.046 | 14.927 | 9.357 |
| mmu-miR-147 | 25.809 | 28.186 | 28.478 | 22.449 |  | 21.933 | 24.234 | 19.808 | 22.093 |
| mmu-miR-148a | 12.334 | 11.968 | 12.593 | 12.241 |  | 11.463 | 11.792 | 12.589 | 11.472 |
| mmu-miR-148b | 15.751 | 15.139 | 15.333 | 18.192 |  | 14.630 | 17.595 | 14.614 | 14.564 |
| mmu-miR-150 | 10.351 | 9.339 | 8.294 | 8.418 |  | 10.874 | 10.288 | 9.500 | 7.723 |
| mmu-miR-151-3p | 11.840 | 11.827 | 11.701 | 11.736 |  | 11.638 | 11.633 | 10.852 | 11.191 |
| mmu-miR-152 | 10.948 | 10.350 | 10.812 | 11.301 |  | 10.271 | 10.317 | 14.369 | 10.434 |
| mmu-miR-153 | 27.569 | 27.184 | 28.478 | 22.746 |  | 22.936 | 24.234 | 20.997 | 20.827 |
| mmu-miR-154 | 21.957 | 22.299 | 22.364 | 18.632 |  | 18.136 | 18.442 | 17.580 | 18.329 |
| mmu-miR-155 | 14.017 | 13.452 | 14.699 | 13.281 |  | 13.997 | 13.749 | 13.082 | 13.588 |
| mmu-miR-15a | 11.980 | 11.287 | 11.518 | 11.029 |  | 11.246 | 12.225 | 11.265 | 11.270 |
| mmu-miR-15b | 10.767 | 10.069 | 10.397 | 9.949 |  | 10.542 | 10.337 | 9.012 | 10.026 |
| mmu-miR-16 | 5.608 | 4.671 | 5.033 | 4.593 |  | 5.363 | 5.255 | 4.947 | 4.806 |
| mmu-miR-17 | 5.565 | 5.690 | 6.740 | 5.982 |  | 5.256 | 5.595 | 6.688 | 6.015 |
| mmu-miR-181a | 10.666 | 10.221 | 9.474 | 9.291 |  | 9.973 | 9.712 | 9.790 | 8.772 |
| mmu-miR-181c | 14.548 | 13.785 | 13.736 | 13.727 |  | 14.817 | 13.474 | 12.523 | 13.118 |
| mmu-miR-182 | 10.172 | 10.261 | 10.801 | 10.956 |  | 9.430 | 9.608 | 10.712 | 10.351 |
| mmu-miR-183 | 12.928 | 13.320 | 13.420 | 13.412 |  | 12.452 | 12.850 | 13.005 | 13.315 |
| mmu-miR-184 | 12.809 | 12.452 | 12.573 | 12.505 |  | 12.757 | 12.788 | 12.445 | 12.116 |
| mmu-miR-185 | 15.820 | 14.745 | 15.430 | 14.681 |  | 13.988 | 14.367 | 12.500 | 13.573 |
| mmu-miR-186 | 10.604 | 9.698 | 9.937 | 11.058 |  | 10.403 | 10.268 | 10.632 | 10.160 |
| mmu-miR-187 | 12.266 | 12.232 | 12.899 | 11.632 |  | 10.852 | 11.336 | 15.163 | 11.691 |
| mmu-miR-188-3p | 27.569 | 28.186 | 28.478 | 23.950 |  | 23.940 | 24.234 | 20.303 | 24.147 |
| mmu-miR-188-5p | 14.750 | 13.627 | 14.414 | 15.588 |  | 14.146 | 13.660 | 12.783 | 13.647 |
| mmu-miR-18a | 11.999 | 11.992 | 13.083 | 13.373 |  | 11.508 | 11.561 | 14.294 | 13.457 |
| mmu-miR-18b | 25.506 | 25.937 | 28.478 | 23.950 |  | 21.939 | 24.234 | 19.427 | 22.398 |
| mmu-miR-190 | 18.304 | 17.464 | 19.080 | 19.314 |  | 18.573 | 19.197 | 15.792 | 19.050 |
| mmu-miR-191 | 5.919 | 5.147 | 4.927 | 4.774 |  | 4.794 | 4.654 | 6.155 | 4.170 |
| mmu-miR-192 | 13.382 | 12.939 | 13.002 | 13.019 |  | 12.800 | 13.114 | 14.533 | 12.172 |
| mmu-miR-193 | 18.975 | 17.549 | 17.132 | 17.300 |  | 19.790 | 18.367 | 14.342 | 16.394 |
| mmu-miR-193b | 8.283 | 8.416 | 8.067 | 8.522 |  | 7.864 | 8.003 | 9.111 | 8.167 |
| mmu-miR-194 | 14.423 | 13.871 | 14.263 | 13.781 |  | 15.609 | 14.816 | 12.218 | 13.461 |
| mmu-miR-195 | 13.700 | 8.545 | 8.779 | 9.846 |  | 10.465 | 11.518 | 11.819 | 9.465 |
| mmu-miR-196b | 19.052 | 17.334 | 19.447 | 20.732 |  | 19.321 | 20.302 | 21.463 | 20.235 |
| mmu-miR-197 | 27.569 | 22.883 | 25.720 | 23.950 |  | 22.206 | 24.234 | 19.150 | 24.147 |
| mmu-miR-199a-3p | 8.760 | 8.156 | 8.791 | 8.311 |  | 8.425 | 8.593 | 9.371 | 7.752 |
| mmu-miR-199a-5p | 14.438 | 14.290 | 14.652 | 17.367 |  | 14.167 | 15.266 | 14.312 | 16.655 |
| mmu-miR-19a | 9.374 | 8.909 | 9.339 | 9.362 |  | 8.543 | 8.868 | 7.845 | 9.185 |
| mmu-miR-19b | 5.304 | 4.864 | 5.383 | 4.712 |  | 4.333 | 4.320 | 5.573 | 4.498 |
| mmu-miR-200a | 10.814 | 9.770 | 9.907 | 9.600 |  | 9.995 | 9.842 | 8.991 | 8.966 |
| mmu-miR-200b | 8.373 | 7.289 | 7.352 | 7.540 |  | 8.287 | 7.534 | 7.244 | 7.141 |
| mmu-miR-200c | 7.526 | 6.850 | 6.358 | 7.343 |  | 7.907 | 7.329 | 11.802 | 6.366 |
| mmu-miR-201 | 27.569 | 28.186 | 28.478 | 23.950 |  | 23.940 | 24.234 | 23.816 | 24.147 |
| mmu-miR-202-3p | 22.587 | 20.356 | 20.844 | 21.525 |  | 21.670 | 19.324 | 23.816 | 22.151 |
| mmu-miR-202-5p | 27.569 | 28.186 | 28.478 | 23.950 |  | 23.940 | 24.234 | 19.614 | 24.147 |
| mmu-miR-203 | 11.486 | 11.172 | 10.900 | 11.724 |  | 11.225 | 11.057 | 11.088 | 10.702 |
| mmu-miR-204 | 11.344 | 11.230 | 11.667 | 11.803 |  | 10.987 | 11.295 | 11.259 | 11.051 |
| mmu-miR-205 | 11.451 | 12.553 | 12.898 | 12.889 |  | 11.304 | 11.400 | 16.016 | 11.528 |
| mmu-miR-207 | 27.569 | 28.186 | 28.478 | 23.950 |  | 23.940 | 24.234 | 22.044 | 24.147 |
| mmu-miR-208 | 20.488 | 18.019 | 20.257 | 19.816 |  | 18.919 | 19.580 | 19.601 | 19.625 |
| mmu-miR-208b | 22.495 | 16.204 | 21.104 | 18.004 |  | 20.373 | 20.210 | 16.165 | 20.445 |
| mmu-miR-20a | 6.528 | 6.710 | 7.611 | 7.122 |  | 6.154 | 6.569 | 7.326 | 6.975 |
| mmu-miR-20b | 8.832 | 8.713 | 9.968 | 9.200 |  | 8.430 | 8.561 | 9.267 | 8.986 |
| mmu-miR-210 | 9.764 | 9.087 | 9.510 | 8.982 |  | 9.379 | 9.073 | 13.294 | 8.189 |
| mmu-miR-211 | 27.569 | 28.186 | 28.478 | 23.950 |  | 23.940 | 24.234 | 19.544 | 24.147 |
| mmu-miR-21 | 13.012 | 11.257 | 11.456 | 12.211 |  | 14.150 | 13.328 | 12.427 | 10.691 |
| mmu-miR-214 | 7.452 | 7.644 | 7.501 | 7.249 |  | 6.877 | 6.843 | 9.936 | 6.219 |
| mmu-miR-215 | 20.263 | 15.811 | 19.916 | 18.434 |  | 19.978 | 20.396 | 22.059 | 20.335 |
| mmu-miR-216a | 27.569 | 28.186 | 26.465 | 23.950 |  | 23.940 | 24.234 | 23.816 | 24.147 |
| mmu-miR-216b | 20.097 | 21.258 | 22.790 | 14.402 |  | 19.709 | 20.838 | 23.816 | 20.782 |
| mmu-miR-217 | 27.569 | 24.586 | 25.238 | 23.950 |  | 22.423 | 22.484 | 15.398 | 24.147 |
| mmu-miR-218 | 9.306 | 8.097 | 7.023 | 8.753 |  | 13.664 | 13.365 | 11.254 | 8.290 |
| mmu-miR-219 | 23.552 | 26.104 | 24.037 | 20.956 |  | 19.641 | 13.448 | 18.078 | 17.717 |
| mmu-miR-220 | 27.569 | 28.186 | 28.478 | 23.950 |  | 23.940 | 24.234 | 19.911 | 20.676 |
| mmu-miR-221 | 13.012 | 12.523 | 12.341 | 16.051 |  | 16.391 | 18.557 | 15.485 | 15.182 |
| mmu-miR-222 | 9.862 | 9.519 | 9.153 | 9.253 |  | 9.997 | 9.969 | 8.799 | 8.970 |
| mmu-miR-223 | 9.697 | 8.681 | 8.261 | 8.607 |  | 8.973 | 9.105 | 9.745 | 7.271 |
| mmu-miR-224 | 15.290 | 14.571 | 14.766 | 14.285 |  | 13.740 | 14.884 | 17.307 | 15.731 |
| mmu-miR-23a | 27.569 | 28.186 | 28.478 | 23.950 |  | 23.940 | 24.234 | 20.233 | 24.147 |
| mmu-miR-23b | 14.615 | 14.339 | 13.934 | 13.145 |  | 15.540 | 15.364 | 12.004 | 12.650 |
| mmu-miR-24 | 6.765 | 5.894 | 5.988 | 5.779 |  | 7.278 | 6.847 | 7.320 | 6.197 |
| mmu-miR-25 | 11.690 | 11.785 | 12.348 | 11.581 |  | 11.846 | 11.288 | 10.276 | 12.256 |
| mmu-miR-26a | 8.282 | 7.559 | 7.540 | 6.716 |  | 8.297 | 8.085 | 8.250 | 6.628 |
| mmu-miR-26b | 11.241 | 10.194 | 10.596 | 10.341 |  | 11.628 | 11.343 | 11.012 | 10.372 |
| mmu-miR-27a | 13.719 | 12.402 | 12.681 | 10.939 |  | 14.611 | 12.280 | 11.284 | 10.925 |
| mmu-miR-27b | 12.274 | 11.715 | 12.133 | 10.366 |  | 11.836 | 11.582 | 11.525 | 10.772 |
| mmu-miR-28 | 11.746 | 11.728 | 12.077 | 11.607 |  | 12.090 | 12.077 | 15.533 | 12.014 |
| mmu-miR-290-3p | 27.569 | 28.186 | 28.478 | 23.950 |  | 23.940 | 24.234 | 22.037 | 24.147 |
| mmu-miR-291a-3p | 22.123 | 20.170 | 20.575 | 20.438 |  | 20.741 | 20.452 | 22.037 | 20.974 |
| mmu-miR-291b-5p | 27.569 | 28.186 | 28.478 | 23.950 |  | 23.940 | 24.234 | 21.891 | 22.849 |
| mmu-miR-292-3p | 18.203 | 18.050 | 18.006 | 19.873 |  | 19.285 | 19.520 | 20.395 | 19.857 |
| mmu-miR-293 | 22.734 | 20.039 | 20.121 | 21.403 |  | 19.826 | 19.355 | 20.811 | 20.151 |
| mmu-miR-294 | 20.091 | 18.870 | 19.738 | 20.926 |  | 20.187 | 19.337 | 21.301 | 20.707 |
| mmu-miR-295 | 20.479 | 19.291 | 20.443 | 20.938 |  | 19.617 | 20.706 | 18.757 | 19.956 |
| mmu-miR-296-3p | 17.340 | 17.017 | 17.627 | 19.028 |  | 16.430 | 18.510 | 16.471 | 18.667 |
| mmu-miR-296-5p | 9.272 | 9.713 | 9.832 | 9.748 |  | 9.300 | 9.568 | 12.227 | 9.734 |
| mmu-miR-297b-5p | 23.617 | 20.917 | 19.595 | 20.854 |  | 21.560 | 20.082 | 21.780 | 22.140 |
| mmu-miR-297c | 24.978 | 22.998 | 23.351 | 19.248 |  | 21.431 | 18.769 | 19.891 | 19.130 |
| mmu-miR-298 | 13.753 | 13.989 | 14.413 | 17.288 |  | 13.078 | 15.276 | 13.356 | 15.755 |
| mmu-miR-29a | 13.520 | 12.349 | 12.559 | 11.308 |  | 13.955 | 12.486 | 13.593 | 11.246 |
| mmu-miR-29b | 21.370 | 20.594 | 20.236 | 18.623 |  | 19.580 | 18.213 | 18.752 | 19.204 |
| mmu-miR-29c | 17.128 | 16.328 | 16.446 | 14.947 |  | 16.066 | 16.387 | 13.962 | 15.750 |
| mmu-miR-301a | 9.322 | 8.415 | 9.176 | 8.160 |  | 8.416 | 8.317 | 7.884 | 8.112 |
| mmu-miR-301b | 12.805 | 8.949 | 9.443 | 8.433 |  | 7.644 | 9.019 | 13.325 | 8.697 |
| mmu-miR-302a | 23.875 | 23.277 | 28.478 | 20.497 |  | 19.801 | 21.054 | 23.816 | 24.147 |
| mmu-miR-302b | 24.513 | 24.120 | 27.366 | 23.199 |  | 20.862 | 22.975 | 23.816 | 22.159 |
| mmu-miR-302c | 27.569 | 28.186 | 28.478 | 23.950 |  | 23.940 | 24.234 | 23.816 | 24.147 |
| mmu-miR-302d | 24.318 | 19.523 | 27.480 | 21.914 |  | 19.431 | 21.630 | 19.302 | 20.625 |
| mmu-miR-30a | 11.119 | 9.938 | 9.869 | 8.587 |  | 11.253 | 10.788 | 8.891 | 8.869 |
| mmu-miR-30b | 6.549 | 5.866 | 5.925 | 4.741 |  | 6.026 | 5.807 | 5.612 | 5.103 |
| mmu-miR-30c | 6.851 | 6.122 | 5.963 | 5.349 |  | 6.568 | 6.304 | 7.043 | 5.535 |
| mmu-miR-30d | 12.972 | 11.961 | 11.781 | 10.676 |  | 13.469 | 12.163 | 10.868 | 10.645 |
| mmu-miR-30e | 10.426 | 9.246 | 9.222 | 7.894 |  | 9.833 | 9.261 | 9.545 | 8.519 |
| mmu-miR-31 | 11.708 | 11.185 | 11.725 | 11.518 |  | 12.314 | 12.332 | 11.757 | 12.537 |
| mmu-miR-320 | 9.148 | 9.170 | 9.200 | 8.331 |  | 8.355 | 8.647 | 7.896 | 8.133 |
| mmu-miR-322 | 11.189 | 8.920 | 8.449 | 7.509 |  | 10.490 | 9.059 | 9.180 | 7.771 |
| mmu-miR-323-3p | 12.798 | 13.468 | 13.589 | 14.665 |  | 12.314 | 13.064 | 15.563 | 17.139 |
| mmu-miR-32 | 17.451 | 16.796 | 16.993 | 14.915 |  | 17.020 | 15.277 | 14.291 | 15.560 |
| mmu-miR-324-3p | 13.185 | 13.305 | 13.372 | 16.660 |  | 16.872 | 15.353 | 13.036 | 13.027 |
| mmu-miR-324-5p | 13.701 | 13.390 | 13.990 | 13.380 |  | 15.013 | 13.623 | 16.782 | 13.131 |
| mmu-miR-325 | 27.569 | 28.186 | 28.478 | 22.202 |  | 23.940 | 24.234 | 19.308 | 24.147 |
| mmu-miR-328 | 9.890 | 9.967 | 9.560 | 9.095 |  | 9.541 | 9.591 | 10.449 | 8.782 |
| mmu-miR-329 | 16.513 | 16.799 | 17.036 | 18.008 |  | 17.811 | 16.857 | 18.710 | 18.150 |
| mmu-miR-330 | 22.856 | 17.951 | 21.352 | 19.659 |  | 18.666 | 19.392 | 17.317 | 18.925 |
| mmu-miR-331-3p | 9.244 | 9.836 | 9.668 | 10.812 |  | 10.170 | 10.433 | 11.626 | 10.525 |
| mmu-miR-331-5p | 18.580 | 17.857 | 17.492 | 19.449 |  | 19.662 | 19.175 | 16.844 | 18.423 |
| mmu-miR-335-3p | 10.542 | 9.686 | 10.372 | 9.705 |  | 9.991 | 9.878 | 9.679 | 9.250 |
| mmu-miR-335-5p | 9.869 | 8.595 | 8.896 | 8.096 |  | 9.086 | 8.543 | 9.506 | 7.847 |
| mmu-miR-337-3p | 15.099 | 14.808 | 15.572 | 14.758 |  | 14.255 | 15.818 | 13.499 | 17.669 |
| mmu-miR-337-5p | 14.409 | 14.331 | 14.494 | 15.096 |  | 14.647 | 14.848 | 16.692 | 16.243 |
| mmu-miR-338-3p | 21.219 | 19.156 | 19.567 | 19.090 |  | 19.039 | 19.107 | 18.015 | 19.435 |
| mmu-miR-339-3p | 13.271 | 12.286 | 12.370 | 12.076 |  | 12.890 | 12.924 | 11.614 | 11.933 |
| mmu-miR-339-5p | 13.590 | 13.519 | 13.625 | 17.132 |  | 16.814 | 17.122 | 16.243 | 17.103 |
| mmu-miR-340-3p | 18.485 | 14.934 | 15.378 | 15.124 |  | 10.809 | 12.841 | 13.767 | 14.813 |
| mmu-miR-340-5p | 17.380 | 12.891 | 13.648 | 12.079 |  | 15.064 | 13.509 | 12.090 | 11.424 |
| mmu-miR-342-3p | 8.013 | 8.648 | 7.796 | 8.061 |  | 12.921 | 8.268 | 16.068 | 7.860 |
| mmu-miR-342-5p | 16.325 | 16.439 | 16.414 | 16.206 |  | 18.583 | 19.181 | 18.981 | 18.858 |
| mmu-miR-344 | 16.076 | 16.187 | 16.588 | 14.901 |  | 15.856 | 18.029 | 17.138 | 17.149 |
| mmu-miR-345-3p | 18.145 | 17.595 | 17.858 | 16.054 |  | 18.800 | 16.606 | 17.716 | 18.435 |
| mmu-miR-345-5p | 14.775 | 14.941 | 15.071 | 13.482 |  | 15.829 | 16.058 | 17.294 | 13.422 |
| mmu-miR-346 | 27.569 | 28.186 | 28.478 | 23.950 |  | 21.585 | 24.234 | 20.175 | 24.147 |
| mmu-miR-34a | 16.818 | 12.731 | 12.934 | 16.538 |  | 16.797 | 16.756 | 16.441 | 16.922 |
| mmu-miR-34b-3p | 13.219 | 11.820 | 10.821 | 10.413 |  | 13.503 | 13.596 | 11.827 | 10.546 |
| mmu-miR-34c | 18.342 | 15.591 | 14.887 | 16.798 |  | 17.416 | 18.126 | 15.759 | 15.252 |
| mmu-miR-350 | 16.590 | 16.035 | 16.538 | 15.347 |  | 16.634 | 15.304 | 13.832 | 17.294 |
| mmu-miR-351 | 9.995 | 9.072 | 9.382 | 9.866 |  | 10.414 | 10.083 | 10.594 | 10.208 |
| mmu-miR-361 | 14.643 | 14.735 | 15.030 | 13.124 |  | 15.134 | 14.390 | 17.694 | 16.122 |
| mmu-miR-362-3p | 17.670 | 16.387 | 17.263 | 15.322 |  | 16.076 | 17.090 | 21.030 | 15.572 |
| mmu-miR-363 | 27.569 | 28.186 | 28.478 | 23.950 |  | 23.940 | 24.234 | 19.558 | 24.147 |
| mmu-miR-365 | 10.950 | 10.653 | 10.221 | 9.166 |  | 10.575 | 10.322 | 14.035 | 9.046 |
| mmu-miR-367 | 27.569 | 24.705 | 28.478 | 23.950 |  | 23.940 | 24.234 | 21.555 | 24.147 |
| mmu-miR-369-3p | 20.185 | 19.237 | 19.463 | 21.933 |  | 19.280 | 19.340 | 18.294 | 19.853 |
| mmu-miR-369-5p | 14.957 | 15.196 | 15.646 | 14.969 |  | 15.221 | 15.381 | 13.786 | 15.586 |
| mmu-miR-370 | 10.585 | 10.808 | 12.089 | 10.663 |  | 10.119 | 10.100 | 10.597 | 10.699 |
| mmu-miR-375 | 11.393 | 11.217 | 11.598 | 11.321 |  | 12.046 | 11.608 | 10.335 | 11.376 |
| mmu-miR-376a | 11.304 | 10.727 | 11.121 | 9.411 |  | 9.387 | 9.699 | 9.820 | 9.096 |
| mmu-miR-376b | 12.290 | 12.147 | 12.805 | 10.989 |  | 15.528 | 11.871 | 13.618 | 11.088 |
| mmu-miR-376c | 12.056 | 11.550 | 11.697 | 12.004 |  | 11.767 | 12.110 | 13.846 | 11.658 |
| mmu-miR-377 | 23.877 | 23.007 | 19.670 | 20.210 |  | 19.786 | 19.978 | 17.310 | 19.827 |
| mmu-miR-379 | 14.139 | 8.920 | 9.656 | 8.798 |  | 9.015 | 8.794 | 11.288 | 8.774 |
| mmu-miR-380-3p | 18.504 | 18.078 | 19.387 | 17.564 |  | 19.145 | 18.247 | 18.140 | 19.270 |
| mmu-miR-380-5p | 13.487 | 13.090 | 14.107 | 12.896 |  | 12.652 | 13.309 | 13.802 | 12.901 |
| mmu-miR-381 | 20.553 | 19.154 | 20.227 | 18.773 |  | 18.324 | 16.498 | 16.779 | 19.128 |
| mmu-miR-382 | 9.849 | 9.227 | 10.050 | 14.887 |  | 15.171 | 15.083 | 16.674 | 15.126 |
| mmu-miR-383 | 15.621 | 16.430 | 16.078 | 15.324 |  | 16.406 | 17.415 | 19.627 | 15.624 |
| mmu-miR-384-3p | 25.988 | 24.364 | 26.467 | 22.195 |  | 19.894 | 22.481 | 21.303 | 22.355 |
| mmu-miR-384-5p | 16.867 | 16.875 | 17.688 | 19.014 |  | 18.314 | 19.136 | 16.545 | 19.185 |
| mmu-miR-409-3p | 7.498 | 7.891 | 8.172 | 9.226 |  | 7.926 | 8.286 | 10.541 | 8.730 |
| mmu-miR-409-5p | 16.753 | 16.461 | 16.527 | 17.494 |  | 17.804 | 18.422 | 16.536 | 17.959 |
| mmu-miR-410 | 10.784 | 10.561 | 10.833 | 10.363 |  | 10.300 | 10.570 | 9.765 | 10.382 |
| mmu-miR-411 | 8.634 | 7.827 | 8.556 | 6.777 |  | 7.407 | 7.391 | 8.775 | 6.758 |
| mmu-miR-423-5p | 14.371 | 15.506 | 15.106 | 15.979 |  | 15.986 | 16.532 | 13.203 | 16.181 |
| mmu-miR-425 | 7.927 | 11.488 | 11.461 | 11.247 |  | 11.876 | 11.001 | 10.401 | 11.100 |
| mmu-miR-429 | 8.801 | 8.147 | 8.195 | 8.107 |  | 8.547 | 8.298 | 7.946 | 8.089 |
| mmu-miR-431 | 8.983 | 8.598 | 9.156 | 12.447 |  | 12.400 | 12.637 | 11.301 | 10.412 |
| mmu-miR-433 | 10.046 | 10.550 | 10.525 | 9.945 |  | 9.896 | 10.272 | 9.668 | 9.676 |
| mmu-miR-434-3p | 10.033 | 9.924 | 9.945 | 9.385 |  | 9.722 | 9.814 | 10.242 | 9.170 |
| mmu-miR-434-5p | 13.582 | 13.389 | 13.716 | 15.347 |  | 12.505 | 16.472 | 13.891 | 11.738 |
| mmu-miR-448 | 26.397 | 28.186 | 28.478 | 22.196 |  | 22.447 | 22.733 | 16.752 | 22.612 |
| mmu-miR-449a | 13.732 | 6.843 | 6.882 | 6.910 |  | 9.414 | 7.580 | 11.757 | 7.051 |
| mmu-miR-449b | 25.566 | 28.186 | 28.478 | 23.950 |  | 23.940 | 24.234 | 19.493 | 24.147 |
| mmu-miR-450a-5p | 11.595 | 14.691 | 14.991 | 15.346 |  | 17.268 | 16.325 | 20.493 | 14.515 |
| mmu-miR-450b-5p | 25.813 | 21.250 | 24.499 | 21.938 |  | 19.779 | 20.176 | 19.511 | 21.768 |
| mmu-miR-451 | 13.221 | 12.436 | 12.135 | 11.023 |  | 11.973 | 12.362 | 15.488 | 11.329 |
| mmu-miR-452 | 27.569 | 28.186 | 28.478 | 23.950 |  | 21.668 | 24.234 | 21.730 | 24.147 |
| mmu-miR-453 | 25.634 | 28.186 | 28.478 | 23.950 |  | 23.940 | 24.234 | 17.890 | 24.147 |
| mmu-miR-455 | 12.452 | 13.178 | 13.463 | 13.077 |  | 14.163 | 14.434 | 17.969 | 13.730 |
| mmu-miR-464 | 27.569 | 28.186 | 28.478 | 23.950 |  | 23.940 | 24.234 | 23.816 | 24.147 |
| mmu-miR-465a-3p | 27.569 | 26.428 | 28.478 | 23.950 |  | 23.940 | 24.234 | 23.816 | 22.890 |
| mmu-miR-465a-5p | 27.569 | 28.186 | 28.478 | 23.950 |  | 23.940 | 24.234 | 23.816 | 24.147 |
| mmu-miR-465b-5p | 27.569 | 28.186 | 28.478 | 23.950 |  | 23.940 | 24.234 | 23.816 | 24.147 |
| mmu-miR-466h | 25.066 | 25.193 | 22.910 | 21.197 |  | 23.940 | 24.234 | 20.336 | 18.056 |
| mmu-miR-467a | 14.390 | 13.981 | 14.250 | 13.791 |  | 13.713 | 13.844 | 14.817 | 14.475 |
| mmu-miR-467b | 15.443 | 14.845 | 14.869 | 15.900 |  | 16.084 | 16.595 | 15.808 | 18.232 |
| mmu-miR-467c | 15.074 | 14.678 | 15.339 | 17.922 |  | 14.747 | 16.358 | 16.283 | 14.244 |
| mmu-miR-467d | 18.161 | 17.092 | 17.111 | 19.076 |  | 18.859 | 18.053 | 19.035 | 19.421 |
| mmu-miR-467e | 16.338 | 16.532 | 16.733 | 16.684 |  | 17.863 | 16.833 | 21.312 | 18.512 |
| mmu-miR-468 | 27.569 | 28.186 | 28.478 | 23.950 |  | 23.940 | 24.234 | 23.816 | 24.147 |
| mmu-miR-469 | 27.569 | 28.186 | 28.478 | 23.950 |  | 23.940 | 24.234 | 23.816 | 24.147 |
| mmu-miR-470 | 27.569 | 28.186 | 28.478 | 23.950 |  | 23.940 | 19.516 | 18.377 | 24.147 |
| mmu-miR-484 | 6.029 | 5.317 | 4.892 | 4.987 |  | 5.884 | 5.596 | 10.604 | 5.347 |
| mmu-miR-486 | 24.810 | 28.186 | 15.560 | 18.242 |  | 23.940 | 24.234 | 23.816 | 21.403 |
| mmu-miR-487b | 23.765 | 23.965 | 24.014 | 23.950 |  | 23.184 | 22.001 | 23.816 | 19.667 |
| mmu-miR-488 | 23.565 | 24.475 | 26.967 | 23.950 |  | 23.940 | 22.482 | 21.141 | 22.384 |
| mmu-miR-489 | 15.936 | 15.231 | 16.267 | 15.248 |  | 15.750 | 15.372 | 17.604 | 15.734 |
| mmu-miR-490 | 19.898 | 17.379 | 17.691 | 18.445 |  | 19.029 | 19.242 | 18.270 | 19.105 |
| mmu-miR-491 | 15.985 | 15.319 | 15.009 | 15.421 |  | 16.820 | 18.045 | 14.286 | 14.839 |
| mmu-miR-493 | 17.103 | 17.112 | 18.277 | 18.633 |  | 16.567 | 18.816 | 17.275 | 17.482 |
| mmu-miR-494 | 12.344 | 12.309 | 13.046 | 11.619 |  | 11.545 | 11.790 | 11.517 | 11.946 |
| mmu-miR-495 | 12.067 | 11.847 | 12.258 | 11.342 |  | 11.053 | 11.595 | 13.032 | 11.496 |
| mmu-miR-496 | 20.728 | 18.267 | 18.218 | 18.786 |  | 18.877 | 19.453 | 18.025 | 19.328 |
| mmu-miR-497 | 15.006 | 13.494 | 13.613 | 12.827 |  | 14.293 | 13.053 | 14.267 | 12.687 |
| mmu-miR-499 | 22.545 | 14.462 | 19.648 | 18.941 |  | 18.430 | 19.388 | 18.556 | 19.178 |
| mmu-miR-500 | 16.555 | 14.855 | 16.269 | 17.515 |  | 18.178 | 17.683 | 17.318 | 17.836 |
| mmu-miR-501-3p | 13.067 | 12.638 | 12.888 | 16.806 |  | 16.355 | 16.917 | 15.775 | 17.219 |
| mmu-miR-503 | 11.938 | 9.707 | 10.202 | 8.068 |  | 10.238 | 9.127 | 12.761 | 8.547 |
| mmu-miR-504 | 19.211 | 15.625 | 25.986 | 18.945 |  | 18.025 | 18.854 | 23.816 | 19.284 |
| mmu-miR-505 | 27.569 | 28.186 | 28.478 | 23.950 |  | 23.940 | 24.234 | 23.816 | 24.147 |
| mmu-miR-509-3p | 27.569 | 26.168 | 27.483 | 22.137 |  | 22.196 | 22.988 | 23.816 | 21.593 |
| mmu-miR-509-5p | 27.569 | 28.186 | 28.478 | 23.950 |  | 23.940 | 24.234 | 21.314 | 24.147 |
| mmu-miR-511 | 25.825 | 20.506 | 17.503 | 19.160 |  | 19.821 | 22.033 | 17.384 | 22.111 |
| mmu-miR-532-3p | 10.998 | 10.978 | 11.105 | 11.800 |  | 11.842 | 11.834 | 11.611 | 11.940 |
| mmu-miR-532-5p | 10.313 | 9.845 | 10.506 | 11.093 |  | 10.969 | 10.830 | 15.049 | 11.013 |
| mmu-miR-539 | 19.529 | 18.479 | 18.979 | 19.538 |  | 18.690 | 19.548 | 23.816 | 19.144 |
| mmu-miR-540-3p | 27.569 | 28.186 | 28.478 | 23.950 |  | 23.940 | 24.234 | 21.528 | 24.147 |
| mmu-miR-540-5p | 22.758 | 19.478 | 20.296 | 19.032 |  | 19.180 | 19.396 | 17.274 | 19.742 |
| mmu-miR-542-3p | 16.514 | 15.343 | 16.335 | 15.887 |  | 16.009 | 16.995 | 16.551 | 16.162 |
| mmu-miR-542-5p | 14.746 | 13.730 | 13.933 | 11.749 |  | 13.820 | 13.068 | 12.292 | 12.302 |
| mmu-miR-543 | 13.403 | 13.699 | 13.919 | 13.778 |  | 14.633 | 13.476 | 15.042 | 16.530 |
| mmu-miR-544 | 17.199 | 16.627 | 16.877 | 17.429 |  | 18.498 | 18.617 | 20.055 | 19.183 |
| mmu-miR-546 | 27.569 | 28.186 | 28.478 | 23.950 |  | 23.940 | 24.234 | 22.073 | 24.147 |
| mmu-miR-547 | 25.336 | 20.148 | 19.674 | 19.537 |  | 22.436 | 21.923 | 19.781 | 19.569 |
| mmu-miR-551b | 23.580 | 19.744 | 19.740 | 18.645 |  | 20.382 | 18.558 | 16.900 | 18.854 |
| mmu-miR-574-3p | 8.725 | 8.420 | 8.130 | 8.286 |  | 8.506 | 8.860 | 11.429 | 8.236 |
| mmu-miR-582-3p | 19.027 | 20.258 | 18.857 | 19.585 |  | 20.351 | 20.249 | 19.701 | 19.148 |
| mmu-miR-582-5p | 23.453 | 17.837 | 18.617 | 19.494 |  | 19.485 | 19.439 | 21.450 | 19.505 |
| mmu-miR-590-5p | 26.090 | 22.326 | 26.737 | 23.950 |  | 23.940 | 24.234 | 21.457 | 24.147 |
| mmu-miR-598 | 20.149 | 18.654 | 22.494 | 19.886 |  | 18.989 | 19.939 | 21.457 | 19.666 |
| mmu-miR-615-3p | 27.569 | 28.186 | 28.478 | 17.982 |  | 23.940 | 24.234 | 23.816 | 24.147 |
| mmu-miR-615-5p | 27.569 | 28.186 | 28.478 | 23.950 |  | 23.940 | 24.234 | 19.557 | 24.147 |
| mmu-miR-652 | 11.705 | 11.505 | 11.328 | 12.464 |  | 12.786 | 13.839 | 16.802 | 12.278 |
| mmu-miR-654-3p | 27.569 | 28.186 | 28.478 | 23.950 |  | 23.940 | 24.234 | 23.816 | 24.147 |
| mmu-miR-654-5p | 27.569 | 28.186 | 28.478 | 23.950 |  | 23.940 | 24.234 | 21.535 | 24.147 |
| mmu-miR-665 | 21.991 | 19.746 | 21.401 | 18.967 |  | 19.098 | 19.401 | 18.186 | 19.583 |
| mmu-miR-666-5p | 14.940 | 14.222 | 13.896 | 15.926 |  | 13.842 | 17.504 | 12.943 | 16.157 |
| mmu-miR-667 | 9.936 | 9.970 | 9.956 | 9.604 |  | 9.805 | 9.774 | 10.750 | 9.561 |
| mmu-miR-668 | 15.859 | 16.242 | 15.468 | 14.742 |  | 17.632 | 14.925 | 17.527 | 14.363 |
| mmu-miR-669a | 15.487 | 14.802 | 14.807 | 15.896 |  | 17.467 | 15.855 | 18.785 | 15.782 |
| mmu-miR-670 | 27.569 | 26.200 | 28.478 | 23.950 |  | 23.940 | 24.234 | 21.055 | 24.147 |
| mmu-miR-671-3p | 15.687 | 15.853 | 16.047 | 18.013 |  | 15.286 | 16.926 | 13.919 | 17.036 |
| mmu-miR-672 | 9.058 | 8.287 | 8.350 | 8.563 |  | 8.935 | 8.344 | 8.912 | 8.560 |
| mmu-miR-674 | 14.235 | 13.985 | 14.118 | 12.797 |  | 13.729 | 13.784 | 13.417 | 13.184 |
| mmu-miR-675-3p | 13.548 | 13.291 | 14.018 | 15.075 |  | 16.765 | 17.032 | 20.429 | 17.547 |
| mmu-miR-675-5p | 27.569 | 28.186 | 28.478 | 23.950 |  | 23.940 | 24.234 | 19.799 | 24.147 |
| mmu-miR-676 | 11.224 | 10.996 | 11.071 | 10.202 |  | 11.272 | 11.073 | 12.168 | 10.543 |
| mmu-miR-677 | 24.079 | 26.685 | 25.225 | 22.445 |  | 18.739 | 22.191 | 18.689 | 20.256 |
| mmu-miR-679 | 20.205 | 18.124 | 21.037 | 19.525 |  | 18.782 | 19.274 | 17.967 | 19.374 |
| mmu-miR-680 | 16.346 | 17.093 | 19.737 | 18.612 |  | 17.385 | 18.837 | 17.020 | 19.036 |
| mmu-miR-682 | 14.296 | 14.732 | 15.262 | 17.115 |  | 15.300 | 16.165 | 16.637 | 17.686 |
| mmu-miR-683 | 27.569 | 28.186 | 28.478 | 23.950 |  | 23.940 | 24.234 | 22.058 | 24.147 |
| mmu-miR-684 | 24.809 | 24.669 | 26.616 | 23.950 |  | 23.940 | 24.234 | 18.315 | 24.147 |
| mmu-miR-685 | 22.295 | 16.431 | 15.804 | 17.467 |  | 18.052 | 18.177 | 20.073 | 18.450 |
| mmu-miR-686 | 27.569 | 28.186 | 28.478 | 23.950 |  | 23.940 | 24.234 | 23.816 | 24.147 |
| mmu-miR-687 | 18.874 | 19.173 | 19.727 | 23.950 |  | 23.940 | 24.234 | 19.687 | 24.147 |
| mmu-miR-708 | 11.540 | 11.467 | 12.027 | 11.586 |  | 10.743 | 10.803 | 15.671 | 11.238 |
| mmu-miR-741 | 27.569 | 26.237 | 28.478 | 19.067 |  | 23.940 | 21.473 | 23.816 | 22.515 |
| mmu-miR-742 | 27.569 | 28.186 | 28.478 | 19.342 |  | 23.940 | 24.234 | 23.816 | 24.147 |
| mmu-miR-743a | 27.569 | 28.186 | 28.478 | 23.950 |  | 23.940 | 24.234 | 23.816 | 24.147 |
| mmu-miR-743b-3p | 27.569 | 28.186 | 28.478 | 23.950 |  | 23.940 | 24.234 | 23.816 | 24.147 |
| mmu-miR-743b-5p | 27.569 | 28.186 | 28.478 | 23.950 |  | 23.940 | 24.234 | 19.805 | 24.147 |
| mmu-miR-744 | 11.241 | 11.577 | 11.776 | 11.161 |  | 11.433 | 11.067 | 12.383 | 11.338 |
| mmu-miR-770-3p | 18.609 | 17.115 | 22.967 | 23.950 |  | 15.880 | 18.800 | 18.900 | 21.647 |
| mmu-miR-7a | 21.370 | 19.823 | 20.778 | 19.817 |  | 19.277 | 20.182 | 19.810 | 19.864 |
| mmu-miR-7b | 18.995 | 18.510 | 19.654 | 19.183 |  | 17.957 | 18.282 | 21.563 | 19.394 |
| mmu-miR-802 | 24.557 | 28.186 | 27.225 | 22.693 |  | 23.940 | 24.234 | 23.816 | 24.147 |
| mmu-miR-871 | 27.569 | 28.186 | 28.478 | 23.950 |  | 23.940 | 24.234 | 20.054 | 24.147 |
| mmu-miR-872 | 12.577 | 12.151 | 12.778 | 12.630 |  | 12.029 | 12.602 | 16.534 | 12.332 |
| mmu-miR-873 | 26.575 | 28.186 | 27.475 | 22.450 |  | 21.935 | 24.234 | 23.816 | 24.147 |
| mmu-miR-874 | 27.569 | 28.186 | 28.478 | 23.950 |  | 23.940 | 24.234 | 23.816 | 24.147 |
| mmu-miR-875-3p | 27.569 | 28.186 | 28.478 | 23.950 |  | 23.940 | 24.234 | 23.816 | 24.147 |
| mmu-miR-876-3p | 27.569 | 28.186 | 28.478 | 23.950 |  | 23.940 | 24.234 | 23.816 | 24.147 |
| mmu-miR-876-5p | 27.569 | 28.186 | 28.478 | 23.950 |  | 23.940 | 24.234 | 23.816 | 24.147 |
| mmu-miR-878-5p | 27.569 | 28.186 | 28.478 | 23.950 |  | 23.940 | 24.234 | 23.816 | 24.147 |
| mmu-miR-879 | 26.028 | 24.529 | 26.975 | 22.693 |  | 22.434 | 24.234 | 23.816 | 24.147 |
| mmu-miR-881 | 27.569 | 28.186 | 28.478 | 23.950 |  | 23.940 | 24.234 | 23.816 | 24.147 |
| mmu-miR-883a-3p | 27.569 | 28.186 | 28.478 | 23.950 |  | 23.940 | 24.234 | 23.816 | 24.147 |
| mmu-miR-883a-5p | 27.569 | 28.186 | 28.478 | 23.950 |  | 23.940 | 24.234 | 23.816 | 24.147 |
| mmu-miR-883b-3p | 27.569 | 28.186 | 28.478 | 23.950 |  | 23.940 | 24.234 | 18.742 | 24.147 |
| mmu-miR-92a | 6.841 | 7.260 | 7.771 | 7.788 |  | 6.998 | 7.266 | 7.724 | 8.033 |
| mmu-miR-93 | 9.513 | 9.602 | 10.120 | 10.086 |  | 9.300 | 9.533 | 11.018 | 10.180 |
| mmu-miR-9 | 15.688 | 14.779 | 16.607 | 18.131 |  | 13.880 | 15.001 | 18.522 | 18.513 |
| mmu-miR-96 | 19.128 | 18.808 | 18.607 | 17.545 |  | 16.923 | 19.161 | 21.316 | 19.035 |
| mmu-miR-98 | 27.569 | 19.060 | 28.478 | 23.950 |  | 22.643 | 24.234 | 19.367 | 24.147 |
| mmu-miR-99a | 10.623 | 10.389 | 11.061 | 10.527 |  | 10.538 | 10.234 | 9.902 | 10.120 |
| mmu-miR-99b | 7.337 | 7.689 | 7.526 | 8.720 |  | 8.103 | 8.154 | 9.559 | 7.675 |
| rno-miR-1 | 12.344 | 10.037 | 14.225 | 11.450 |  | 12.330 | 13.857 | 16.260 | 12.222 |
| rno-miR-17-3p | 27.569 | 26.816 | 26.973 | 22.959 |  | 23.940 | 24.234 | 21.629 | 24.147 |
| rno-miR-190b | 19.086 | 17.891 | 19.416 | 19.204 |  | 19.075 | 19.485 | 18.873 | 19.555 |
| rno-miR-196c | 12.885 | 15.461 | 16.699 | 17.522 |  | 14.818 | 16.534 | 17.059 | 16.951 |
| rno-miR-207 | 27.569 | 24.926 | 28.478 | 23.950 |  | 21.186 | 21.461 | 20.811 | 21.391 |
| rno-miR-20b-3p | 27.569 | 28.186 | 28.478 | 23.950 |  | 23.940 | 24.234 | 23.816 | 24.147 |
| rno-miR-219-1-3p | 20.085 | 18.255 | 18.526 | 18.971 |  | 18.882 | 18.429 | 23.816 | 19.077 |
| rno-miR-219-2-3p | 27.569 | 26.685 | 28.478 | 23.950 |  | 23.940 | 22.487 | 20.418 | 24.147 |
| rno-miR-224 | 14.541 | 13.831 | 14.196 | 13.318 |  | 13.363 | 14.032 | 16.906 | 14.065 |
| rno-miR-327 | 27.569 | 28.186 | 28.478 | 19.570 |  | 23.940 | 24.234 | 23.816 | 24.147 |
| rno-miR-333 | 27.569 | 28.186 | 28.478 | 23.950 |  | 23.940 | 24.234 | 23.816 | 24.147 |
| rno-miR-336 | 27.569 | 28.186 | 28.478 | 23.950 |  | 23.940 | 24.234 | 20.391 | 24.147 |
| rno-miR-339-3p | 14.753 | 13.706 | 13.207 | 13.284 |  | 13.399 | 12.899 | 12.431 | 12.468 |
| rno-miR-343 | 27.569 | 28.186 | 26.479 | 20.803 |  | 23.940 | 24.234 | 20.126 | 24.147 |
| rno-miR-344-3p | 27.569 | 28.186 | 28.478 | 23.950 |  | 23.940 | 24.234 | 23.816 | 24.147 |
| rno-miR-344-5p | 27.569 | 26.184 | 28.478 | 23.950 |  | 18.819 | 22.232 | 20.620 | 24.147 |
| rno-miR-345-3p | 14.991 | 14.633 | 15.217 | 14.159 |  | 15.067 | 14.325 | 17.612 | 14.227 |
| rno-miR-346 | 27.569 | 28.186 | 25.993 | 23.950 |  | 23.940 | 24.234 | 23.816 | 24.147 |
| rno-miR-347 | 27.569 | 28.186 | 28.478 | 23.950 |  | 23.940 | 24.234 | 23.816 | 24.147 |
| rno-miR-349 | 27.569 | 28.186 | 28.478 | 23.950 |  | 23.940 | 24.234 | 19.747 | 24.147 |
| rno-miR-351 | 9.664 | 8.951 | 9.522 | 10.905 |  | 11.230 | 10.581 | 14.739 | 10.981 |
| rno-miR-377 | 25.814 | 26.439 | 25.219 | 22.068 |  | 23.940 | 22.471 | 20.463 | 22.645 |
| rno-miR-381 | 16.069 | 15.525 | 15.913 | 16.265 |  | 15.537 | 17.733 | 19.463 | 15.743 |
| rno-miR-409-5p | 27.569 | 28.186 | 28.478 | 23.950 |  | 23.940 | 24.234 | 23.816 | 24.147 |
| rno-miR-421 | 27.569 | 28.186 | 28.478 | 23.950 |  | 23.940 | 24.234 | 20.753 | 24.147 |
| rno-miR-450a | 16.504 | 14.840 | 14.911 | 14.318 |  | 17.998 | 18.052 | 17.504 | 17.962 |
| rno-miR-466b | 27.569 | 28.186 | 28.478 | 23.950 |  | 23.940 | 24.234 | 23.816 | 24.147 |
| rno-miR-466c | 24.163 | 22.648 | 25.729 | 20.081 |  | 20.876 | 22.588 | 23.816 | 21.891 |
| rno-miR-505 | 27.569 | 28.186 | 28.478 | 23.950 |  | 23.940 | 24.234 | 23.816 | 24.147 |
| rno-miR-532-5p | 19.940 | 11.142 | 19.975 | 15.687 |  | 19.990 | 20.191 | 23.816 | 16.066 |
| rno-miR-543 | 27.569 | 28.186 | 25.715 | 23.950 |  | 23.940 | 24.234 | 23.816 | 24.147 |
| rno-miR-598-5p | 26.151 | 22.814 | 22.934 | 23.950 |  | 21.788 | 21.618 | 23.816 | 23.144 |
| rno-miR-673 | 22.880 | 24.216 | 22.172 | 19.244 |  | 22.192 | 22.487 | 23.816 | 20.387 |
| rno-miR-742 | 27.569 | 28.186 | 28.478 | 23.950 |  | 23.940 | 24.234 | 23.816 | 24.147 |
| rno-miR-743b | 27.569 | 28.186 | 28.478 | 23.950 |  | 23.940 | 19.698 | 21.189 | 24.147 |
| rno-miR-758 | 17.019 | 16.717 | 17.319 | 18.835 |  | 16.082 | 16.090 | 18.947 | 19.245 |
| rno-miR-760-5p | 27.569 | 28.186 | 28.478 | 23.950 |  | 23.940 | 24.234 | 21.574 | 24.147 |
| rno-miR-871 | 27.569 | 28.186 | 28.478 | 23.950 |  | 23.940 | 24.234 | 23.816 | 24.147 |
| rno-miR-878 | 27.569 | 28.186 | 28.478 | 23.950 |  | 23.940 | 24.234 | 23.816 | 24.147 |
| rno-miR-881 | 27.569 | 28.186 | 28.478 | 23.950 |  | 23.940 | 24.234 | 19.057 | 24.147 |
| snoRNA135 | 7.402 | 8.098 | 8.190 | 9.487 |  | 8.498 | 8.355 | 8.159 | 8.771 |
| snoRNA202 | 4.731 | 5.122 | 5.165 | 9.115 |  | 6.643 | 5.812 | 6.211 | 5.718 |
| U87 | 13.778 | 10.110 | 9.937 | 9.165 |  | 14.603 | 11.176 | 10.752 | 13.558 |
| Y1 | 11.599 | 11.260 | 12.643 | 15.293 |  | 16.689 | 16.933 | 17.145 | 16.789 |
