# Supplementary figures and images for: miRNA regulated pathways in late stage murine lung development
Source: BMC Dev Biol. 2013 Apr 24;13:13. doi: 10.1186/1471-213X-13-13 (PMC3644234; doi:10.1186/1471-213X-13-13)

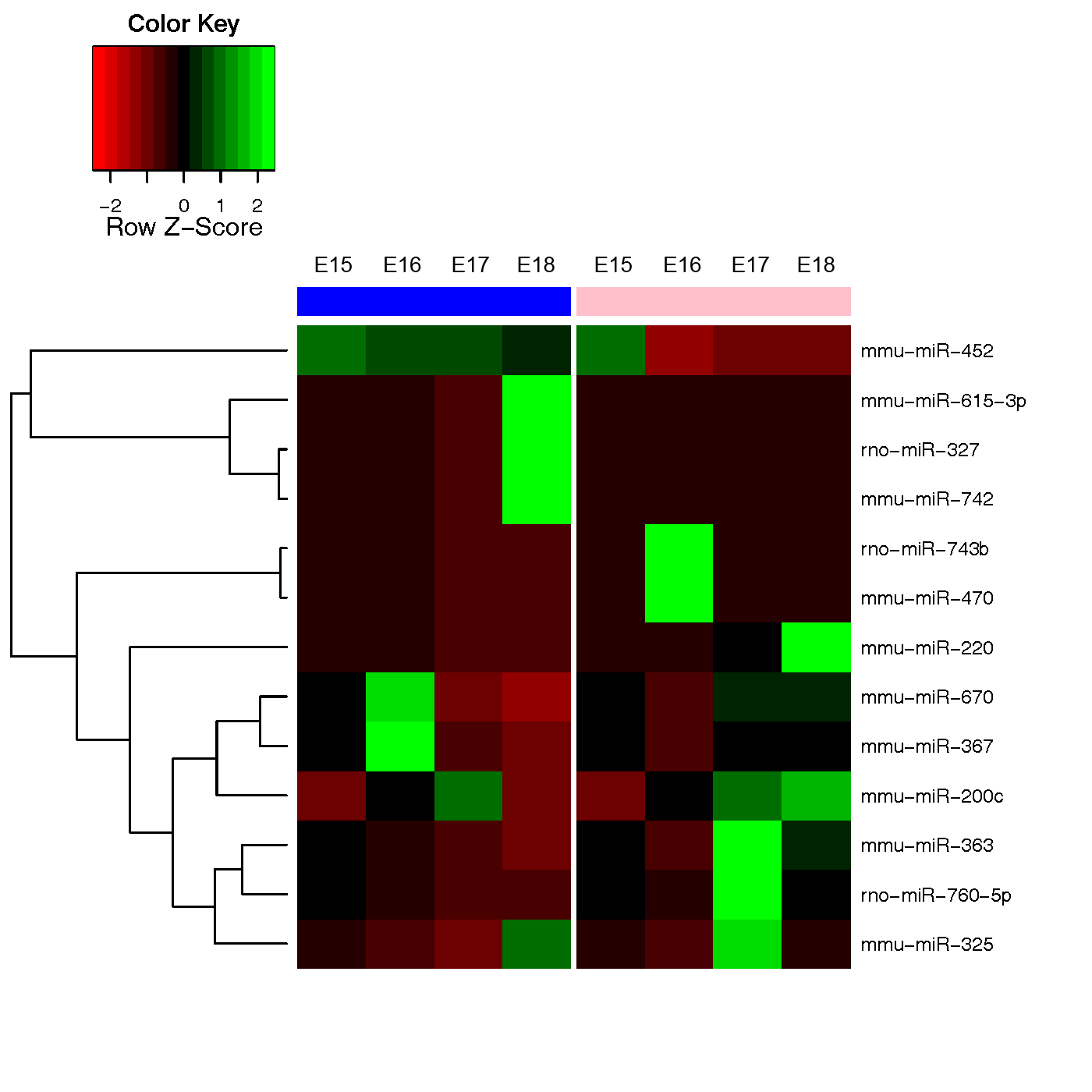

Supplement: Additional file 2 — Figure:Expression patterns of miRNAs that changed significantly with sex and gestation. Total RNA was isolated from male and female E15 – E18 whole lungs and miRNA expression profiling was done using Taqman Rodent miRNA real-time PCR array. Columns with blue bars indicated male lungs, and columns with pink bars indicate female lungs. Each gestational day is normalized to the male E15 time point. mmu-miR-363 and mmu-miR-325 are the only miRNAs that are exclusive to this interaction group. The rest of the miRNAs in this group overlap with those present in the sex and gestation groups. [file 1471-213X-13-13-S2.tiff]
