# Supplementary material for: miRNA regulated pathways in late stage murine lung development
Source: BMC Dev Biol. 2013 Apr 24;13:13. doi: 10.1186/1471-213X-13-13 (PMC3644234; doi:10.1186/1471-213X-13-13)
Supplement: Additional file 3 — Table:List of miRNA regulators and targets. Using the Ingenuity Pathway Analysis database, the GO Annotations, upstream regulators, and downstream regulators/targets of miRNAs that changed significantly between sexes and with gestation were identified. [file 1471-213X-13-13-S3.docx]

| **miRNA** | **M15** | **M16** | **M17** | **M18** | **F15** | **F16** | **F17** | **F18** |
| --- | --- | --- | --- | --- | --- | --- | --- | --- |
| *Gender* | Fold Change values | | | | Fold Change values | | | |
| mmu-miR-802 | 1 | 0.08 | 6.36 | 3.64 | 1 | 0.82 | 1.09 | 1.15 |
| mmu-miR-138 | 1 | 1.25 | 2.01 | 0.63 | 1 | 0.78 | 0.45 | 2.03 |
| mmu-miR-182 | 1 | 0.94 | 1.55 | 0.58 | 1 | 0.88 | 0.41 | 1.89 |
| mmu-miR-296-3p | 1 | 1.25 | 1.22 | 0.31 | 1 | 0.24 | 0.97 | 4.72 |
| mmu-miR-125a-5p | 1 | 0.82 | 0.88 | 0.38 | 1 | 0.83 | 0.04 | 0.76 |
| mmu-miR-532-3p | 1 | 1.01 | 1.08 | 0.57 | 1 | 1.01 | 1.17 | 1.07 |
| mmu-miR-652 | 1 | 1.15 | 0.77 | 0.59 | 1 | 0.48 | 0.06 | 0.70 |
| mmu-miR-455 | 1 | 0.60 | 2.01 | 0.65 | 1 | 0.83 | 0.07 | 0.74 |
| mmu-miR-670 | 1 | 2.58 | 1.88 | 12.29 | 1 | 0.82 | 7.38 | 1.15 |
| mmu-miR-367 | 1 | 7.28 | 1.88 | 12.29 | 1 | 0.82 | 5.22 | 1.15 |
| rno-miR-207 | 1 | 6.25 | 1.88 | 12.29 | 1 | 0.83 | 1.30 | 1.15 |
| mmu-miR-31 | 1 | 1.44 | 1.01 | 1.14 | 1 | 0.99 | 1.47 | 1.17 |
| mmu-miR-351 | 1 | 1.90 | 0.65 | 1.09 | 1 | 1.26 | 0.88 | 0.87 |
| rno-miR-351 | 1 | 1.64 | 0.91 | 0.42 | 1 | 1.57 | 0.09 | 0.84 |
| mmu-miR-220 | 1 | 0.65 | 1.88 | 12.29 | 1 | 0.82 | 16.32 | 0.10 |
| mmu-miR-219 | 1 | 0.17 | 1.40 | 6.04 | 1 | 73.15 | 2.95 | 0.26 |
| mmu-miR-24 | 1 | 1.83 | 0.58 | 1.98 | 1 | 1.35 | 0.97 | 0.47 |
| mmu-miR-141 | 1 | 2.89 | 0.42 | 4.68 | 1 | 1.74 | 3.15 | 0.32 |
| rno-miR-743b | 1 | 0.65 | 1.88 | 12.29 | 1 | 18.92 | 6.73 | 1.15 |
| mmu-miR-470 | 1 | 0.65 | 1.88 | 12.29 | 1 | 21.46 | 47.27 | 1.15 |
| mmu-miR-615-3p | 1 | 0.65 | 1.88 | 769.35 | 1 | 0.82 | 1.09 | 1.15 |
| rno-miR-327 | 1 | 0.65 | 1.88 | 255.82 | 1 | 0.82 | 1.09 | 1.15 |
| mmu-miR-742 | 1 | 0.65 | 1.88 | 299.73 | 1 | 0.82 | 1.09 | 1.15 |
| mmu-miR-486 | 1 | 0.10 | 0.00 | 94.88 | 1 | 0.82 | 1.09 | 0.17 |
|  |  |  |  |  |  |  |  |  |
| *Gestation* |  |  |  |  |  |  |  |  |
| mmu-miR-452 | 1 | 0.65 | 1.88 | 12.29 | 1 | 0.17 | 0.96 | 5.57 |
| mmu-miR-147 | 1 | 0.19 | 6.36 | 10.26 | 1 | 0.20 | 4.36 | 1.12 |
| mmu-miR-504 | 1 | 12.00 | 109.53 | 1.20 | 1 | 0.56 | 0.02 | 2.39 |
| rno-miR-743b | 1 | 0.65 | 1.88 | 12.29 | 1 | 18.92 | 6.73 | 1.15 |
| mmu-miR-470 | 1 | 0.65 | 1.88 | 12.29 | 1 | 21.46 | 47.27 | 1.15 |
| mmu-miR-409-3p | 1 | 0.76 | 1.60 | 0.30 | 1 | 0.78 | 0.16 | 1.75 |
| mmu-miR-92a | 1 | 0.75 | 1.90 | 0.52 | 1 | 0.83 | 0.60 | 2.05 |
| mmu-miR-17 | 1 | 0.92 | 2.26 | 0.75 | 1 | 0.79 | 0.37 | 1.69 |
| mmu-miR-18a | 1 | 1.01 | 2.12 | 0.39 | 1 | 0.96 | 0.14 | 3.86 |
| mmu-miR-670 | 1 | 2.58 | 1.88 | 12.29 | 1 | 0.82 | 7.38 | 1.15 |
| mmu-miR-367 | 1 | 7.28 | 1.88 | 12.29 | 1 | 0.82 | 5.22 | 1.15 |
| rno-miR-760-5p | 1 | 0.65 | 1.88 | 12.29 | 1 | 0.82 | 5.15 | 1.15 |
| mmu-miR-325 | 1 | 0.65 | 1.88 | 41.28 | 1 | 0.82 | 24.78 | 1.15 |
| mmu-miR-220 | 1 | 0.65 | 1.88 | 12.29 | 1 | 0.82 | 16.32 | 0.10 |
| mmu-miR-351 | 1 | 1.90 | 0.65 | 1.09 | 1 | 1.26 | 0.88 | 0.87 |
| mmu-miR-200c | 1 | 1.60 | 0.45 | 1.13 | 1 | 1.49 | 0.07 | 0.34 |
| mmu-miR-484 | 1 | 1.64 | 0.45 | 2.06 | 1 | 1.22 | 0.04 | 0.69 |
| mmu-miR-24 | 1 | 1.83 | 0.58 | 1.98 | 1 | 1.35 | 0.97 | 0.47 |
| mmu-miR-126-3p | 1 | 2.51 | 0.39 | 1.67 | 1 | 1.66 | 1.19 | 0.28 |
| mmu-miR-26a | 1 | 1.65 | 0.60 | 2.96 | 1 | 1.16 | 1.03 | 0.31 |
| mmu-miR-30e | 1 | 2.27 | 0.43 | 5.78 | 1 | 1.49 | 1.22 | 0.40 |
| mmu-miR-322 | 1 | 4.82 | 0.15 | 12.81 | 1 | 2.69 | 2.48 | 0.15 |
| mmu-miR-146a | 1 | 3.18 | 0.17 | 3.39 | 1 | 1.45 | 2.34 | 0.19 |
| mmu-miR-150 | 1 | 2.02 | 0.24 | 3.82 | 1 | 1.50 | 2.59 | 0.11 |
| mmu-miR-34b-3p | 1 | 2.64 | 0.19 | 6.99 | 1 | 0.94 | 3.20 | 0.13 |
| mmu-miR-29a | 1 | 2.25 | 0.51 | 4.63 | 1 | 2.77 | 1.29 | 0.15 |
| mmu-miR-30d | 1 | 2.01 | 0.44 | 4.91 | 1 | 2.47 | 6.06 | 0.14 |
| mmu-miR-146b | 1 | 2.31 | 0.31 | 3.54 | 1 | 1.84 | 0.13 | 0.17 |
| mmu-miR-27a | 1 | 2.49 | 0.49 | 6.87 | 1 | 5.03 | 10.04 | 0.08 |
| mmu-miR-30a | 1 | 2.27 | 0.42 | 5.78 | 1 | 1.38 | 5.14 | 0.19 |
| mmu-miR-139-3p | 1 | 20.73 | 0.00 | 379.37 | 1 | 15.79 | 235.00 | 0.00 |
| mmu-miR-615-3p | 1 | 0.65 | 1.88 | 769.35 | 1 | 0.82 | 1.09 | 1.15 |
| rno-miR-327 | 1 | 0.65 | 1.88 | 255.82 | 1 | 0.82 | 1.09 | 1.15 |
| mmu-miR-742 | 1 | 0.65 | 1.88 | 299.73 | 1 | 0.82 | 1.09 | 1.15 |
| mmu-miR-486 | 1 | 0.10 | 0.00 | 94.88 | 1 | 0.82 | 1.09 | 0.17 |
| mmu-miR-340-5p | 1 | 22.46 | 0.08 | 39.43 | 1 | 2.94 | 7.86 | 0.08 |
| mmu-miR-449a | 1 | 118.57 | 0.01 | 113.15 | 1 | 3.56 | 0.20 | 0.19 |
