# Supplementary material for: miRNA regulated pathways in late stage murine lung development
Source: BMC Dev Biol. 2013 Apr 24;13:13. doi: 10.1186/1471-213X-13-13 (PMC3644234; doi:10.1186/1471-213X-13-13)
Supplement: Additional file 4 — Fold change values of miRNAs that changed significantly between sexes and gestation. Fold change values were calculated using deltaCT values in Additional file 1. [file 1471-213X-13-13-S4.docx]

| **miRNA gene** | **GO Annotation (Biological Process)** | **Upstream Regulators** | **Downstream regulators or Targets** |
| --- | --- | --- | --- |
| *Gender* |  |  |  |
| mmu-miR-802 | cellular response to amino acid stimulus |  | N/A |
| mmu-miR-138 | cellular response to growth factor stimulus; myelination | DICER1, PASMC cells, EIF2C2, EPHB6, perchlorate, methimazole, BRAF | VIM, CCND3 |
| mmu-miR-182 | Wnt receptor signaling pathway | CARD11, MALAT1, INHBC, NLGN2, GJA3, NCAM1, NFASC, CASP12, FOXO1, CASP2, COL4A4, MTSS1, COL11A2, CDK6, SOS1 | RASA1, GRB2 |
| mmu-miR-296-3p |  | TSH, tretinoin |  |
| mmu-miR-125a-5p |  | capecitabine, PASMC cells, EIF2C2, EPHB6, VPR, 4-hydroxynonenal, docetaxel, metribolone, Insulin, hydrogen peroxide, TP53, tretinoin, lipopolysaccharide, acetaminophen | TP53, MAZ, CCNA2, E2F3, BAK1, CBFB |
| mmu-miR-532-3p |  | TCF4 |  |
| mmu-miR-652 | cellular response to inorganic substance | Influenza A virus strain A/TX/36/91, Influenza A virus (A/Brevig Mission/1/1918(H1N1)) |  |
| mmu-miR-455 | cellular response to amino acid stimulus | PASMC cells, EIF2C2, TNFSF12 |  |
| Y1 |  |  |  |
| mmu-miR-670 |  |  |  |
| mmu-miR-367 | lung development | INHBA, TNF |  |
| rno-miR-207 | cellular response to estrogen stimulus | acetaminophen | DDX20 |
| mmu-miR-31 | cellular response to amino acid stimulus; cellular response to inorganic substance | 5-fluorouracil, TNF, decitabine, human herpesvirus 8, E2F1, E2F3, hydrogen peroxide, TGFB1, trichostatin A, RAN, Xpo5, XPO5, Ran, Erk | FOXP3, XPO5, Fmrp, DICER1, Xpo5, TIAM1 |
| rno-miR-351 | cellular response to amino acid stimulus; cellular response to inorganic substance | hydrogen peroxide |  |
| mmu-miR-351 | cellular response to amino acid stimulus; cellular response to inorganic substance | hydrogen peroxide |  |
| mmu-miR-220 |  |  |  |
| mmu-miR-219 | cellular response to growth factor stimulus; negative regulation of cell proliferation; negative regulation of gene expression; positive regulation of oligodendrocyte differentiation; regulation of myelination | resolvin D1, STAT3, zymosan A | DDX20, ZNF238, FOXJ3, SOX6, PDGFRA, ICOS |
| mmu-miR-24 | cellular response to lipopolysaccharide; myelination; positive regulation of angiogenesis; regulation of viral genome replication; retina vasculature development in camera-type eye | EIF2C2, PASMC cells, EPHB6, vorinostat, 5-fluorouracil, arsenic trioxide, TM cells, trichostatin A, decitabine, Insulin, valproic acid, lithium, E2F3, E2F1, beta-estradiol | DDX20, FAF1, EIF2C2, PASMC cells, EPHB6, vorinostat, 5-fluorouracil, arsenic trioxide, TM cells, trichostatin A, decitabine, Insulin, valproic acid, lithium, E2F3, E2F1, beta-estradiol |
| mmu-miR-141 | cellular response to amino acid stimulus; cellular response to glucose stimulus; gene silencing by miRNA; olfactory bulb interneuron differentiation; regulation of insulin secretion involved in cellular response to glucose stimulus; Wnt receptor signaling pathway | 5-fluorouracil, human herpesvirus 4 strain B95-8, progesterone, Bcr, EIF2C2, EIF2C1, GRHL2, PKD1, TAT, VPR, vorinostat, docetaxel, 25-hydroxy-vitamin D3, ibandronic acid, cisplatin | ZEB1, ZEB2, CDH1, HDAC4, FTH1, DLX5, BMRF1, Bzlf1, GEMIN2, TGFB2, STAT5B, Hif, CTNNB1, CCNE2 |
| rno-miR-743b |  |  |  |
| mmu-miR-470 | negative regulation of gene expression | TNFSF12 | DDX20 |
| mmu-miR-615-3p |  | Insulin | LCOR, PPARG |
| rno-miR-327 |  |  |  |
| mmu-miR-742 |  |  |  |
| mmu-miR-486 | cellular response to amino acid stimulus; cellular response to estrogen stimulus; cellular response to lipopolysaccharide | tretinoin, TP53 |  |
| *Gestation* |  |  |  |
| mmu-miR-452 |  | human herpesvirus 8 |  |
| mmu-miR-147 | BMP signaling pathway; negative regulation of inflammatory response | TAT, VPR, 5-fluorouracil, TLR4 |  |
| mmu-miR-504 |  | CTGF | FOXP1 |
| rno-miR-743b |  |  |  |
| mmu-miR-470 | negative regulation of gene expression | TNFSF12 | DDX20 |
| mmu-miR-409-3p |  | vorinostat |  |
| mmu-miR-92a |  | 5-fluorouracil, PASMC cells, EIF2C2, vorinostat, oxaliplatin, 25-hydroxy-vitamin D3, Map2k1/2, TRBP, decitabine, trichostatin A, E2F3, TP53, hydrogen peroxide, E2F1 | BCL2L11, TP63, IKZF1, FBXW7, CDK7, CCND2, CCNE1, PTEN, CCND1, bromodeoxyuridine, ZEB2, CDKN1A, IKZF1, FBXW7, HIPK3, CDKN1C, MAP2K4, ENPP6, BMPR2, VSNL1, MYLIP, ITGA5, EIF2C2 |
| mmu-miR-17 |  | 5-fluorouracil, MYC, E2F1, DICER1, PASMC cells, EIF2C2, EPHB6, VPR, RUNX1, vorinostat, docetaxel, oxaliplatin, 25-hydroxy-vitamin D3, App, Hct 116 cells | E2F1, CDKN1A, RB1, BCL2L11, APP, RUNX1, CCND1, Bhrf1, Lmp-1, E2F3, E2F2, TP63, PTEN, MICA, PKD2, BCL2L11, PTEN, TGFBR2, CREB1, SMAD6/7, PKD2, BNIP2, H2AFX, RUNX2, BMP2, STAT3, ITGB8, CDK7, CCND2, CCNE1 |
| mmu-miR-18a |  |  | KRAS |
| mmu-miR-670 |  |  |  |
| mmu-miR-367 | lung development | INHBA, TNF |  |
| rno-miR-760-5p |  |  |  |
| mmu-miR-325 |  | VPR |  |
| mmu-miR-220 |  |  |  |
| mmu-miR-351 | cellular response to amino acid stimulus; cellular response to inorganic substance | hydrogen peroxide |  |
| mmu-miR-200c | cellular response to amino acid stimulus; cellular response to glucose stimulus; gene silencing by miRNA; olfactory bulb interneuron differentiation; regulation of insulin secretion involved in cellular response to glucose stimulus; Wnt receptor signaling pathway | 5-fluorouracil, human herpesvirus 4 strain B95-8, progesterone, Bcr, EIF2C2, EIF2C1, GRHL2, PKD1, TAT, VPR, vorinostat, docetaxel, 25-hydroxy-vitamin D3, ibandronic acid, cisplatin | ZEB1, ZEB2, CDH1, HDAC4, FTH1, DLX5, BMRF1, Bzlf1, GEMIN2, TGFB2, STAT5B, Hif, CTNNB1, CCNE2 |
| mmu-miR-484 | cellular response to lipopolysaccharide | D-glucose |  |
| mmu-miR-24 | cellular response to lipopolysaccharide; myelination; positive regulation of angiogenesis; regulation of viral genome replication; retina vasculature development in camera-type eye | EIF2C2, PASMC cells, EPHB6, vorinostat, 5-fluorouracil, arsenic trioxide, TM cells, trichostatin A, decitabine, Insulin, valproic acid, lithium, E2F3, E2F1, beta-estradiol | DDX20, FAF1, EIF2C2, PASMC cells, EPHB6, vorinostat, 5-fluorouracil, arsenic trioxide, TM cells, trichostatin A, decitabine, Insulin, valproic acid, lithium, E2F3, E2F1, beta-estradiol |
| mmu-miR-126-3p |  | TCF4, PASMC cells, EIF2C2, vorinostat, TMEM8B, cisplatin, Insulin, TP53, ETS2, ETS1 | IRS1, CRKL, VEGFA, TOM1, VCAM1, PIK3R2, SPRED1 |
| mmu-miR-26a |  | acetaminophen, PASMC cells, EIF2C2, EPHB6, docetaxel, 25-hydroxy-vitamin D3, TP53, E2F1, E2F2, E2F3, hydrogen peroxide, tamoxifen, exemestane, Estrogen receptor, beta-estradiol | EZH2, SMAD1, BAK1, PHF6, MAP2, TGFBR2, PLAG1, CCNE2, CDKN2A, CDK6, CDK4, CCND3, MYC, EPHA2, PGR, CDKN1A, GSK3B, PTEN, CCND2, Hif |
| mmu-miR-30e |  | TCF4, EPHB6, vorinostat, docetaxel, Insulin | FMR1, VEZT, SLC7A6, AQP4, CYR61, TMEM2, TUBA1A, WDR82, THBS1, CDK6 |
| mmu-miR-322 | cellular response to inorganic substance | c-Src, docetaxel, Gulo, hydrogen peroxide, TP53, TNFSF12, beta-estradiol, monocrotaline, phorbol myristate acetate | CCNE1, MAP2K1, CDC25A, CDK2 |
| mmu-miR-146a | cellular response to estrogen stimulus; cellular response to lipopolysaccharide; myelination; regulation of gene expression; response to oxygen levels | TNF, lipopolysaccharide, IL1B, NFkB, phorbol myristate acetate, PASMC cells, EIF2C2, decitabine, palmitic acid, human herpesvirus 4, TMEM8B, herpes simplex virus type-1 strain 17syn+, TNFSF12, resolvin D1 | IL6, IL1B, TNF, Hemoglobin, Immunoglobulin, IL10, IFNG, Cytokine, IL8, Insulin, Tlr, FAF1, Il1, MMP2, TRAF6, IRAK2, IRAK1, FADD |
| mmu-miR-150 | cellular response to lipopolysaccharide; homeostasis of number of cells; immunoglobulin production; regulation of B cell differentiation; regulation of gene expression | DICER1, Plasmodium berghei str. ANKA, BRAF, megakaryocytes, hematopoietic progenitor cells, PRDM5, poly rI:rC-RNA | MYB, Igm, IGA, Igg2b, Igg1, NOTCH3, Immunoglobulin, AKT2, DKC1, ACTA2, IFNG, Surfactant, Collagen Type I, CASP3 |
| mmu-miR-34b-3p |  | TAT, EPHB6, VPR, TP53, nutlin-3a, Wi 38 cells, decitabine |  |
| mmu-miR-29a |  | PASMC cells, EIF2C2 |  |
| mmu-miR-30d |  | TCF4, EPHB6, vorinostat, docetaxel, Insulin | FMR1, VEZT, SLC7A6, AQP4, CYR61, TMEM2, TUBA1A, WDR82, THBS1, CDK6 |
| mmu-miR-146b |  | NFkB, lipopolysaccharide, TNF, TAT, PASMC cells, EIF2C2, VPR, tretinoin, human herpesvirus 4, TMEM8B, cisplatin, LDL, hepatitis C virus subtype 1b, resolvin D1, zymosan A | NFkB, P38 MAPK, MAPK14, Erk, PXN, PTK2B, Jnk, FAK, LYN, HCK, LCK, FGR, FYN, YES1, SRC, CFH, TLR4, KIF22, METTL7A, COL13A1, VWCE, POLE2, MCM10, PBLD, PEX11G, MMP16, BLMH, CDKN3, RAD54L, TRIM14 |
| mmu-miR-27a |  | PPP2R2C, PASMC cells, EIF2C2, EPHB6, 5-fluorouracil, docetaxel, SPI1, Gulo, Plasmodium berghei str. ANKA, TNFSF12, decitabine, trichostatin A, Insulin, E2F2, E2F1 | FBXW7, EPHB4, EFNB2, MYH7, SPRY2, agar, PPARG, FBXW7, THRB, SRM, SMAD5, SMAD4, GCA, RUNX1, MMP13, PEX7, CTNNBIP1, FADD, ST14, PHB, SMAD3 |
| mmu-miR-30a |  | TCF4, EPHB6, vorinostat, docetaxel, Insulin | FMR1, VEZT, SLC7A6, AQP4, CYR61, TMEM2, TUBA1A, WDR82, THBS1, CDK6 |
| mmu-miR-139-3p |  | tretinoin |  |
| mmu-miR-615-3p |  | Insulin | LCOR, PPARG |
| rno-miR-327 |  |  |  |
| mmu-miR-742 |  |  |  |
| mmu-miR-486 | cellular response to amino acid stimulus; cellular response to estrogen stimulus; cellular response to lipopolysaccharide | tretinoin, TP53 |  |
| mmu-miR-340-5p |  |  |  |
| mmu-miR-449a | regulation of gene expression | MECOM, TAT, VPR, TMEM8B | MECOM, agar |
